# Tmbim5 and Slc8b1 cooperate in tissue-specific mitochondrial calcium regulation in zebrafish

**DOI:** 10.1038/s42003-025-09494-7

**Published:** 2026-01-08

**Authors:** Iga Wasilewska, Łukasz Majewski, Dobrochna Adamek-Urbańska, Sofiia Baranykova, Paulina Castañeda-Tamez, Ilka Wittig, Matylda Macias, Aleksandra Szybińska, Axel Methner

**Affiliations:** 1 Mossakowski Medical Research Institute, Polish Academy of Sciences, Warsaw, Poland; 2 Institute for Molecular Medicine, University Medical Center of the Johannes Gutenberg-University Mainz, Mainz, Germany; 3 International Institute of Molecular and Cell Biology in Warsaw, Warsaw, Poland; 4 Department of Ichthyology and Biotechnology in Aquaculture, Institute of Animal Sciences, Warsaw University of Life Sciences, Warsaw, Poland; 5 Institute for Cardiovascular Physiology, Goethe University Frankfurt, Frankfurt am Main, Germany

**Keywords:** Mitochondria, Calcium signalling

## Abstract

Mitochondrial calcium homeostasis involves coordinated uptake via the mitochondrial calcium uniporter (MCU) and efflux through sodium-dependent NCLX (encoded by *SLC8B1*) and/or TMEM65. We investigated TMBIM5, a proposed bidirectional mitochondrial calcium/proton transporter, by generating zebrafish lacking *tmbim5*, *slc8b1*, plus *tmbim5/mcu* and *tmbim5/slc8b1* double knockouts. Tmbim5-deficient fish exhibited growth impairment, muscle atrophy, and increased brain cell death. *tmbim5/mcu* double knockouts showed no additive effects, arguing against Tmbim5 functioning as an independent calcium uptake pathway. *slc8b1* knockouts had no major phenotype but showed attenuated, although not abolished sodium-dependent mitochondrial calcium efflux. *tmbim5/slc8b1* double knockouts showed altered mitochondrial calcium handling with reduced uptake and efflux. Remarkably, brain phenotypes were rescued while muscle dysfunction was exacerbated in double mutants, corresponding to restored mitochondrial membrane potential in brain tissue and decreased calcium levels in muscle. These findings suggest that TMBIM5 functions as an auxiliary calcium efflux pathway cooperating with NCLX in a tissue-specific manner.

## Abstract (web summary)

Tmbim5 and Slc8b1 cooperate in tissue-specific mitochondrial Ca2+ regulation in zebrafish. Genetic analyses show that Tmbim5 is not an independent uptake pathway but acts as an auxiliary Ca2+ efflux mechanism with distinct roles in brain and muscle.

## Introduction

Mitochondria generate ATP by harnessing proton and electron gradients established by the electron transport system. The negative mitochondrial membrane potential across the inner mitochondrial membrane also serves as the driving force for uptake of the universal cellular messenger calcium (Ca^2+^). Increased mitochondrial Ca^2+^ levels enhance oxidative phosphorylation, leading to increased ATP production. Additionally, mitochondria function as cellular Ca^2+^ sinks, making mitochondrial Ca^2+^ uptake crucial for cellular Ca^2+^ signaling. Therefore, mitochondrial Ca^2+^ homeostasis represents a finely tuned process involving the coordinated action of Ca^2+^ transporters, regulatory proteins, and interactions with the endoplasmic reticulum (ER).

The mechanisms governing mitochondrial Ca^2+^ transport remain incompletely understood. The mitochondrial Ca^2+^ channel mediating inward Ca^2+^ flux is the mitochondrial Ca^2+^ uniporter (MCU)^1,2^, which forms part of a large protein complex containing several MCU-interacting and regulatory proteins, including MICU1 (mitochondrial uptake 1)^3^, MICU2^4^, EMRE (essential MCU regulator)^5^, and MCUR1 (mitochondrial Ca^2+^ uniporter regulator 1)^6,7^. Despite its central role, *Mcu* knockout (KO) produces remarkably mild phenotypes: in mice, *Mcu* deletion causes mild phenotypes in a CD1 background^8^ but embryonic lethality in C57Bl/6^9^. In zebrafish, *mcu* knockdown or knockout had minimal effects on survival, activity, viability, fertility, morphology, or swimming behavior, although it abolished mitochondrial Ca^2+^ uptake similar to the well-characterized MCU inhibitor ruthenium red (RuR)^10–13^. In vivo studies in zebrafish photoreceptors suggest alternative Ca^2+^ entry pathways can compensate for Mcu loss^14^.

One disputed candidate for alternative Ca^2+^ transport is Leucine Zipper And EF-Hand Containing Transmembrane Protein 1 (LETM1), identified by genome-wide screening as a RuR-sensitive mitochondrial Ca^2+^/H^+^-antiporter^15^. Originally, LETM1 was described as part of the mitochondrial K^+^/H^+^ exchanger^16^. For Ca^2+^ efflux, the primary mediator is the Na^+^/Ca^2+^/Li^+^ exchanger NCLX^17^, whose knockout causes embryonic lethality in mice^18^. However, the precise transport mechanism of NCLX remains debated. Although long considered a Na⁺-dependent Ca²⁺ efflux transporter, recent structural analyses failed to identify a Na⁺ binding site and instead suggested a pH-dependent mechanism^19^. Recent evidence suggests that Transmembrane Protein 65 (TMEM65) is also involved in Na^+^-dependent Ca^2+^ efflux from mitochondria^20–22^ and may act as a positive regulator of NCLX^23^. Another mitochondrial Ca^2+^ extrusion system is the mitochondrial permeability transition pore (mPTP), a non-selective high-conductance pore of debated identity^24,25^. High mitochondrial Ca^2+^ concentrations trigger mPTP opening, which when sustained causes mitochondrial swelling, breakdown of mitochondrial membrane potential, and eventually cell death (reviewed in ref. ^26^).

Recently, transmembrane BAX inhibitor motif containing protein 5 (TMBIM5) has emerged as a novel candidate for mitochondrial Ca^2+^ transport. TMBIM5 was initially linked to regulation of mitochondrial morphology, as its loss resulted in mitochondrial fragmentation and disrupted cristae structure^27–30^. Loss of TMBIM5 also increased cellular sensitivity to apoptosis^27,28,31^. In vivo, mice with a specific alteration (D326R) that presumably disrupts the channel pore of TMBIM5^32^ and leads to protein degradation^29^ exhibit higher embryonic mortality and develop skeletal myopathy characterized by reduced muscle strength. These observations correlated with several mitochondrial abnormalities, most dramatically in skeletal muscle mitochondria, including disrupted cristae structure, premature mPTP opening, decreased mitochondrial Ca^2+^ uptake capacity, and mitochondrial swelling^29^.

Two independent studies demonstrated that TMBIM5, when reconstituted in liposomes, can function as a Ca^2+^ transporter in the presence of a pH gradient^30,31^. Inhibiting NCLX-mediated mitochondrial Ca^2+^ efflux with CGP-37157^30^ or *NCLX* knockdown^31^ resulted in diminished mitochondrial Ca^2+^ retention capacity in *TMBIM5* KO human embryonic kidney (HEK) cells and reduced RuR-induced mitochondrial Ca^2+^ efflux^30^. *TMBIM5* KO cells also accumulated more matrix Ca^2+^ upon ER Ca^2+^ release mediated by inhibition of the sarco/endoplasmic reticulum ATPase (SERCA) with thapsigargin^31^ or stimulation of inositol trisphosphate receptor-mediated Ca^2+^ release with ATP or carbachol^30^. Restricting mitochondrial Ca^2+^ uptake by concurrent *MCU* knockout rescued elevated apoptosis induced by staurosporine in *TMBIM5*^−/−^ cells^31^. Collectively, these data suggest that TMBIM5 can mediate Ca^2+^ extrusion from mitochondria and that its function at least partially overlaps with NCLX.

In contrast, own previous work found that TMBIM5 overexpression in HEK cells enhanced mitochondrial Ca^2+^ uptake following ER Ca^2+^ release^29^, suggesting that TMBIM5 may operate bidirectionally under certain conditions. This study also demonstrated that under steady-state conditions, loss of TMBIM5 increases potassium and reduces proton levels in the mitochondrial matrix, suggesting a broader role in mitochondrial ion exchange^29^, consistent with a potential interaction between TMBIM5 and LETM1^30^. However, the physiological significance of these mechanisms in vivo, particularly the crosstalk between TMBIM5, MCU, and NCLX, remains unclear.

Due to their translucency, zebrafish represent an ideal model to study mitochondrial Ca^2+^ dynamics in vivo^33,34^. In the present study, we generated *tmbim5* and *slc8b1* KO fish using CRISPR/Cas9-mediated gene editing^35^ and observed that Tmbim5 loss results in a muscle phenotype associated with delayed hatching, reduced size, and increased brain cell death. To investigate potential interactions with known Ca^2+^ transport systems, we generated *tmbim5*/*mcu* and *tmbim5*/*slc8b1* double knockouts. Both lines remained viable without major phenotypes; however, *tmbim5*/*slc8b1* double knockouts showed tissue-specific rescue or aggravation of phenotypes associated with Tmbim5 loss of function. These findings suggest that mitochondrial Ca^2+^ transport systems operate in a highly tissue-specific manner in fish.

## Results

### Loss of Tmbim5 impairs zebrafish development and muscle function

**Fig. 1:**
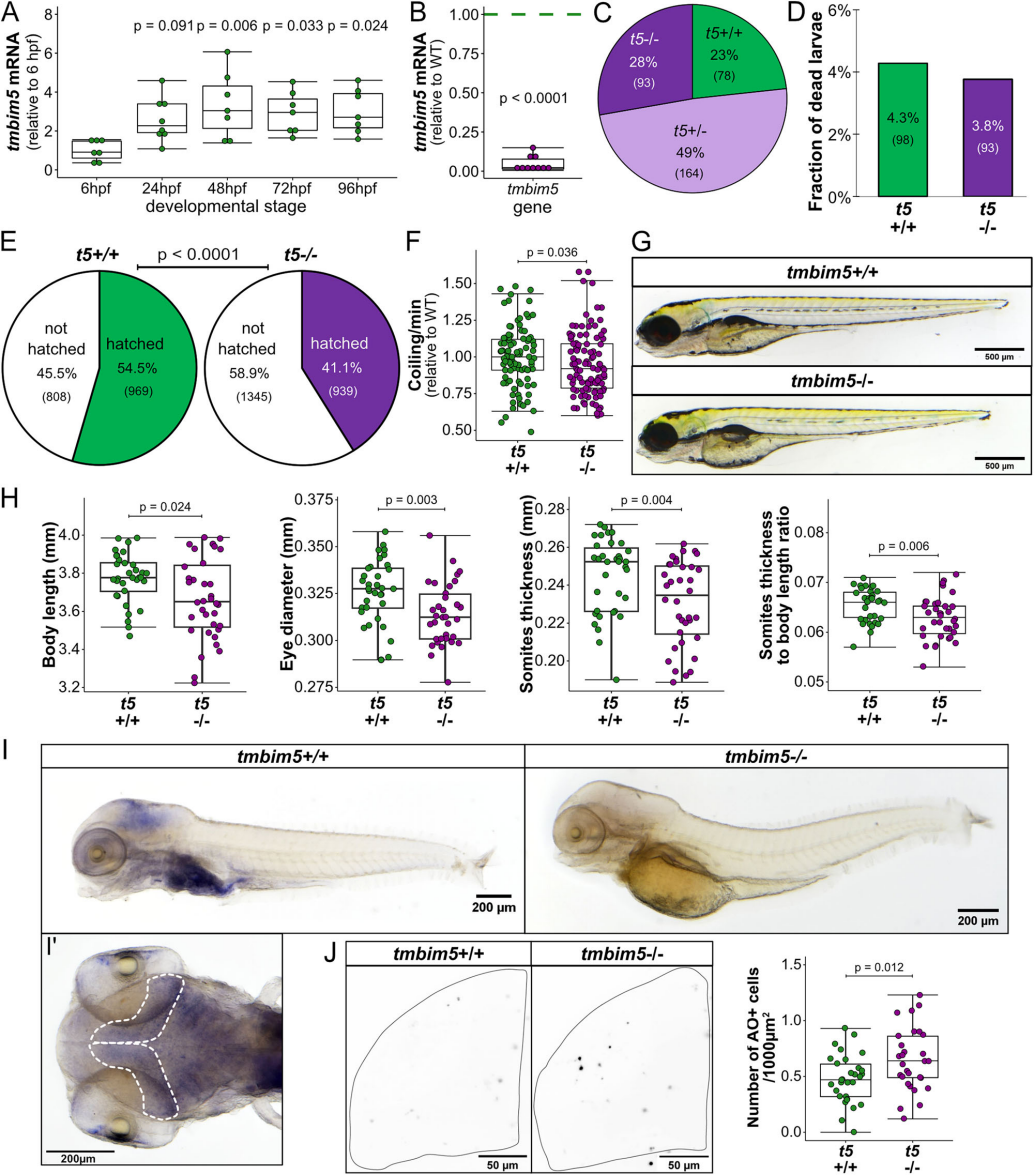
Loss of Tmbim5 impairs zebrafish development and muscle function. **A** *tmbim5* mRNA levels at different developmental stages, measured using quantitative PCR (qPCR). Expression was normalized to 6 h post-fertilization (hpf), with *18S* used as a reference gene. Data are presented as box-and-whisker plots, where the box represents the 25th–75th percentile, and the whiskers indicate the minimum and maximum values. Each dot represents an independent biological replicate (*n* = 7–8; each RNA sample was isolated from 30 embryos/larvae). Number of experiments = 4. Statistical analysis: one-way ANOVA followed by Tukey’s HSD test; *p*-values indicate comparisons with 6 hpf. **B** Decreased *tmbim5* mRNA levels in 5 days post-fertilization (dpf) *tmbim5*^−/−^ larvae, quantified using qPCR and normalized to WT levels. *rpl13a* and *ef1a* were used as reference genes. Data are shown as box-and-whisker plots. Each dot represents an independent biological replicate (*n* = 10; each RNA sample was isolated from 30 larvae). Number of experiments = 5. Statistical analysis: one-sample *t*-test. **C** Genotype distribution of offspring from *tmbim5* (*t5*)^+/-^ zebrafish breeding does not deviate from expected Mendelian ratios (*n* indicated in brackets). Number of experiments = 4. Statistical analysis: Chi-square test. **D** Survival of *tmbim5*^−/−^ larvae up to 5 dpf is not significantly affected. *n* is indicated in brackets. Number of experiments = 7. Statistical analysis: Chi-square test. **E** Reduced hatching efficiency of *tmbim5*^−/−^ larvae. *n* is indicated in brackets. Number of experiments = 6. Statistical analysis: Chi-square test. **F** Decreased coiling activity of *tmbim5*^−/−^ embryos at 1 dpf. The mean number of coiling movements per minute, normalized to WT from the same experiment, is shown as box-and-whisker plots. Each dot represents an individual embryo (*n* = 94–104). Number of experiments = 6. Statistical analysis: one-sample Wilcoxon test. **G** Normal morphology of *tmbim5*^−/−^ larvae at 5 dpf. Representative images of WT (*tmbim5*^+/+^) and *tmbim5*^−/−^ larvae are shown. **H** Reduced size of *tmbim5*^−/−^ larvae. Morphometric analysis results are presented as box-and-whisker plots, with each dot representing an individual larva (*n* = 34–36). Number of experiments = 3. Statistical analysis: Mann–Whitney test (for body length and somite thickness) or *t*-test. **I** *tmbim5* transcript detected using whole-mount in situ hybridization in 4 dpf wild-type (WT) larvae, with a complete loss of expression in *tmbim5*^−/−^ larvae. I’ Dorsal view of the optic tectum region outlined with a dashed line. Number of experiments = 4. **J** Increased cell death in the optic tectum of *tmbim5*^*−/−*^ larvae detected using Acridine Orange (AO) staining. Representative images of WT and *tmbim5*^−/−^ larvae (black dots indicate dying cells with high AO signals). Quantification results are presented as box-and-whisker plots, with each dot representing an individual larva (*n* = 29–30, number of experiments = 3). Statistical analysis: *t*-test.

The zebrafish genome contains only one *TMBIM5* orthologue which is expressed as early as 6 h post fertilization (hpf) and plateaus after 24 h (Fig. 1A). To study the effect of Tmbim5 deficiency, we generated mutant fish by introducing a deletion in exon 4 using CRISPR/Cas9 genome editing^35^. This resulted in a profound decrease in *tmbim5* mRNA (Fig. 1B). Mutants (*tmbim5*^−/−^) reached adulthood and the Mendelian distribution was unchanged (Fig. 1C), indicating no increased embryonic lethality (Fig. 1D).

Mutant larvae took longer to hatch than wild-type (WT, *tmbim5*^+/+^) controls (Fig. 1E) and this correlated with reduced tail coiling (Fig. 1F), spontaneous movements of the tail that enable the embryo to escape from its chorion^36^. This suggested a possible reduction in muscle strength as also observed in mice with a channel pore mutation in TMBIM5^29^. Despite that, mutant larvae displayed unchanged spontaneous locomotor activity in an open field test (Supplementary Fig. 1A) as well as after stimulating mobility in a visual-motor response test (Supplementary Fig. 1B).

Tmbim5-deficient larvae were morphologically normal (Fig. 1G) but shorter, had a smaller eye diameter and thinner somites – segments of trunk and tail of larvae (Fig. 1H). This reduction in somite thickness was not solely attributable to the overall reduction in whole-body size, as the ratio of somite thickness to whole-body length was also decreased in *tmbim5*^−/−^ larvae (Fig. 1H). Using whole-mount in situ hybridization we found that *tmbim5* is strongly expressed in the digestive system, gills, eyes and head with an especially high signal in the area of the midbrain of zebrafish larvae (Fig. 1I). Importantly no staining was observed in *tmbim5*^−/−^ larvae proving the specificity of the riboprobe. Based on the strong expression of *tmbim5* in the brain, we quantified cell death in this tissue by staining the brain of *tmbim5*^−/−^ larvae with Acridine Orange. This demonstrated increased cell death (Fig. 1J) caused by Tmbim5 deficiency. Together these results suggest a role for Tmbim5 in body size, muscle strength and cell viability in the nervous system.

### Tmbim5 deficiency causes muscle atrophy with preferential effects on slow-twitch fibers

**Fig. 2:**
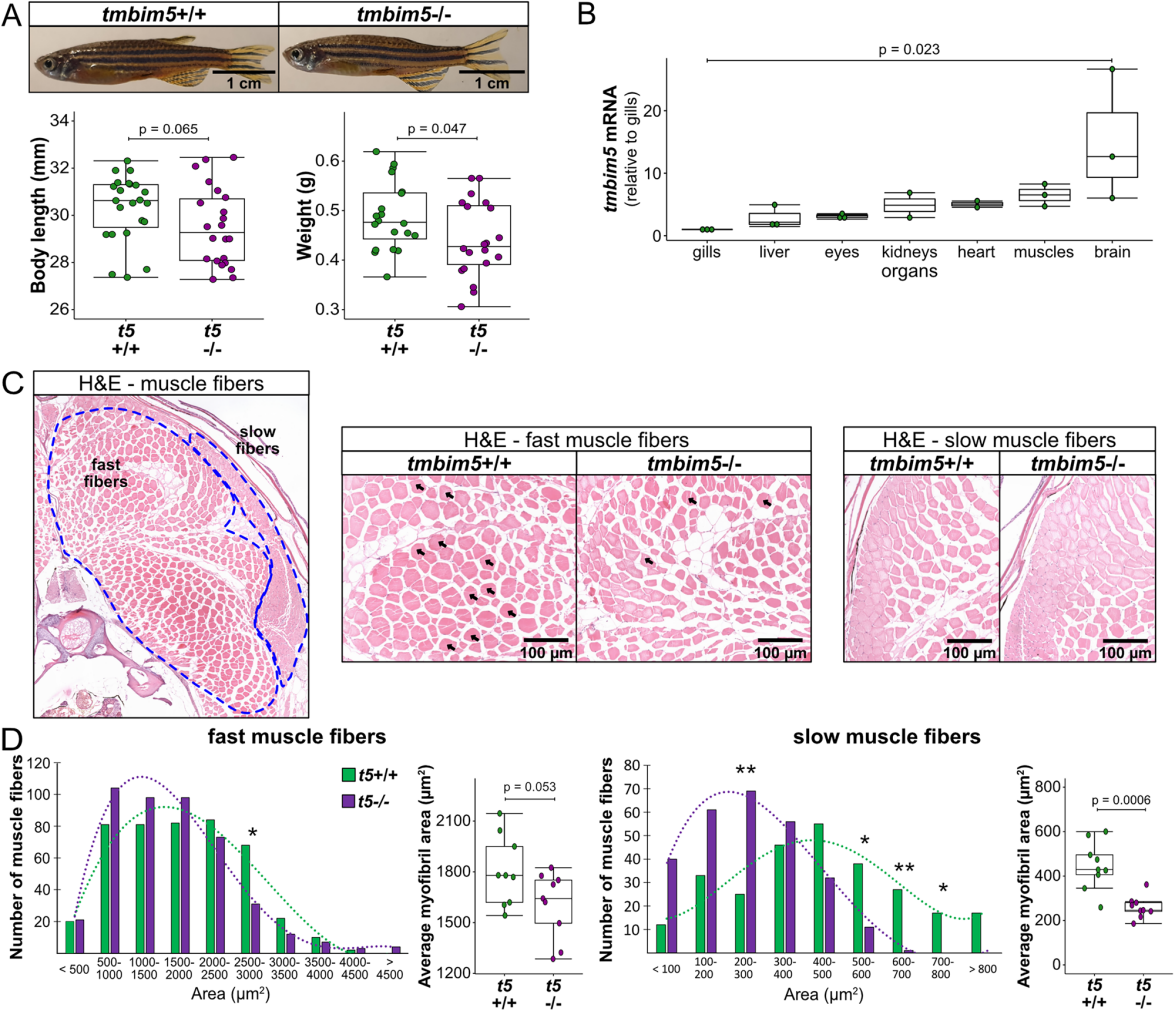
Tmbim5 deficiency causes muscle atrophy with preferential effects on slow-twitch fibers. **A** Smaller size of *tmbim5*^−/−^ adult fish. Representative images of WT and *tmbim5*^−/−^ 8-month-old zebrafish. Morphometric analysis results are presented as box-and-whisker plots (box: 25th–75th percentile; whiskers: min to max), with each dot representing an individual fish (*n* = 19–23). Number of experiments = 3 (weight) or 4 (body length). Statistical analysis: Mann–Whitney test (body length) or *t*-test (weight). **B** *tmbim5* mRNA levels in various adult zebrafish organs measured using qPCR. Expression was normalized to the gills (the organ with the lowest *tmbim5* expression), with *rpl13a* used as a reference gene. Data are presented as box-and-whisker plots, with each dot representing an independent biological replicate (*n* = 2–3; each RNA sample was isolated from 2 fish). Number of experiments = 3. Statistical analysis: Kruskal-Wallis test followed by Dunn’s test. **C** Representative images of hematoxylin and eosin (H&E)-stained skeletal muscle sections from adult (8-month-old) zebrafish with fast- and slow-twitch fibers indicated. Middle panel: magnification of the area with fast fibers images from WT and *tmbim5*^−/−^. Arrows indicate fibers of 2500-3000 µm², which are significantly reduced in number in the mutants. Right panel: magnification of the area with slow-twitch fibers images of WT and *tmbim5*^−/−^. **D** Smaller cross-sectional area of fast and slow muscle fibers in *tmbim5*^−/−^ adult fish. Histograms showing the distribution of fast (left panel) and slow (right panel) muscle fiber cross-sectional areas in WT and *tmbim5*^−/−^ fish. The mutant distribution is shifted toward smaller fibers. Statistical analysis: Chi-square test (fast fibers: *p* = 0.0009; slow fibers: *p* < 0.0001). Differences in the number of fibers of specific sizes are marked with an asterisk (* *p* < 0.05), analyzed using the Mann–Whitney test or *t*-test with Benjamini-Hochberg (BH) correction for multiple comparisons. Box-and-whisker plots showing the average myofibril area. Each dot represents the average for one section (50 fibers analyzed per section, *n* = 9). Number of fish = 3. Statistical analysis: *t*-test.

We next studied the effects of Tmbim5 deficiency in adult zebrafish to determine whether the developmental phenotypes persist into adulthood. Similar to larvae, *tmbim5*^−/−^ adult fish had a normal morphology, a tendency to be smaller and a significant reduction in weight compared to WT fish (Fig. 2A). Locomotor activity of adult fish was unchanged (Supplementary Fig. 1C). Similar to mouse *Tmbim5*, zebrafish *tmbim5* is expressed in most tissues with the highest mRNA expression levels in brain and muscle (Fig. 2B). A histopathological examination of these tissues and liver revealed no major differences (Supplementary Fig. 2A–J).

Because of the muscle phenotype implied by reduced tail coiling and delayed hatching of larvae, we next analyzed the skeletal muscle in more detail. In zebrafish slow (red) and fast (white) twitch fibers are segregated, allowing analysis without immunohistochemical staining^37^ (Fig. 2C). Slow twitch fibers are rich in mitochondria and depend more on aerobic metabolism than fast (white) twitch muscle fibers^38^. A morphometric analysis of these fibers demonstrated a shift towards smaller fibers, a decline in the fibers with a cross-sectional area of 2500–3000 µm^2^, and a reduction in the average cross-sectional area in mutant fish. All of these changes were more pronounced in slow twitch muscle fibers than in fast twitch fibers (Fig. 2D). The more pronounced atrophy of slow-twitch muscle fibers possibly reflects mitochondrial dysfunction and is consistent with the reduced muscle strength observed in larvae.

### Reduced complex I activity and increased antioxidant capacity but unchanged steady-state mitochondrial Ca^2+^ levels, normal ATP and ROS levels in tmbim5^−/−^ larvae

**Fig. 3:**
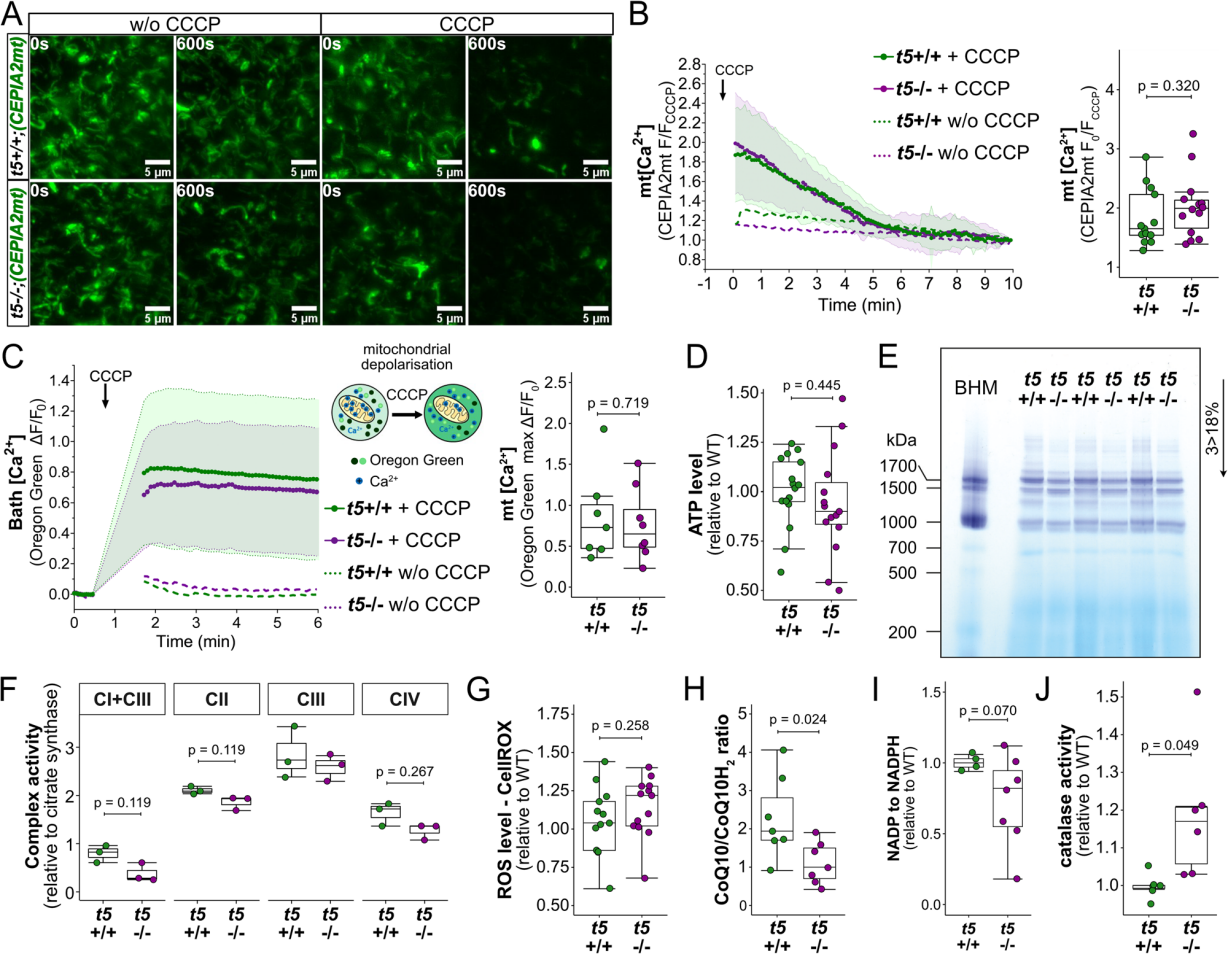
Reduced complex I activity and increased antioxidant capacity but unchanged steady-state mitochondrial Ca^2+^ levels, normal ATP and ROS levels in *tmbim5*^*−/−*^ larvae. **A**, **B** No alteration in basal mtCEPIA2 signal intensity or mitochondrial Ca^2+^ response to depolarization with CCCP. CCCP (10 µM) was used to depolarize mitochondria, and the F_0_/F_CCCP_ ratio was calculated. Larvae incubated in E3 medium without CCCP (w/o CCCP) served as negative controls. **A** Representative images of the region of interest (ROI) at the beginning and after 10 min of CCCP treatment. **B** Left panel: F/F_CCCP_ mean ± SD traces. Right panel: mitochondrial Ca^2+^ efflux level after CCCP treatment calculated as F_0_/F_CCCP_. Data presented as box-and-whisker plots (box: 25th–75th percentile; whiskers: min to max), with each dot representing an individual larva (*n* = 13–14). Statistical analysis: Mann–Whitney test. **C** No alteration in mitochondrial Ca^2+^ levels was observed in isolated mitochondria. CCCP (10 µM) was used to depolarize mitochondria and induce Ca²⁺ efflux. Fluorescence was normalized to the initial value (F_0_). Data are presented as mean ± SD ΔF/F_0_ traces. Mitochondria treated with a buffer without CCCP (w/o CCCP) served as negative controls. Maximal ΔF/F_0_ values were calculated and presented as box-and-whisker plots (box: 25th–75th percentile; whiskers: min to max), with each dot representing an independent biological replicate (*n* = 7–8). Statistical analysis: *t*-test. **D** Normal ATP levels in the samples obtained from *tmbim5*^−/−^ 5 dpf larvae. Data are normalized to average WT levels and presented as box-and-whisker plots, where the box represents the 25th–75th percentile, and the whiskers indicate the minimum and maximum values. Each dot represents an independent biological replicate (*n* = 15–17; each sample was isolated from 10 larvae). Number of experiments = 4. Statistical analysis: *t*-test. **E** Diminished activity of complex I assessed by in-gel activity assay. Digitonin-solubilized isolated mitochondria from 5 dpf wild-type (*t5* + /+) and *tmbim5*^−/−^ (*t5*−/−) were subjected to blue native gel electrophoresis (BNGE) followed by in-gel complex I activity assay. Bovine heart mitochondria (BHM) were used as a positive control (*n* = 3, each sample contained 100 µg of protein). **F** Complex I + III, complex II, complex III and complex IV activity determined by spectrophotometric assay and normalized to citrate synthase activity. Each dot represents an independent biological replicate (*n* = 3). Statistical analysis: *t*-test or Mann–Whitney test with Benjamini-Hochberg (BH) correction for multiple comparisons. **G** No significant difference in the reactive oxygen species (ROS) levels detected using CellROX staining in the brain of WT and *tmbim5*^−/−^ larvae. Data are normalized to average WT levels. Each dot represents an individual larva (*n* = 13). Number of experiments = 3. Statistical analysis: *t*-test. **H** Loss of Tmbim5 resulted in a reduced CoQ10 to CoQ10H_2_ ratio in *tmbim5*^−/−^ larvae determined by High-Performance Liquid Chromatography with Electrochemical Detection (HPLC-ECD). Each dot represents an independent biological replicate (*n* = 7-8, each sample was isolated from 50 larvae). Number of experiments = 4. Statistical analysis: *t*-test. **I** NADP to NADPH ratio in *tmbim5*^−/−^ larvae. Data are normalized to average WT levels. Each dot represents an independent biological replicate (*n* = 4-7, each sample was isolated from 100 larvae). Number of experiments = 3. Statistical analysis: one sample *t*-test. **J** Elevated catalase activity in *tmbim5*^−/−^ larvae. Data are normalized to average WT levels. Each dot represents an independent biological replicate (*n* = 6, each sample was isolated from 100 larvae). Number of experiments = 3. Statistical analysis: one sample *t*-test.

To clarify whether changes in the mitochondrial Ca^2+^ homeostasis could explain the effects of Tmbim5 deficiency, we took advantage of the translucency of zebrafish larvae which allows the quantification of (mitochondrial) Ca^2+^ levels in the living animal using genetically-encoded Ca^2+^ sensors. We crossed tmbim5 mutant with fish *Tg*(*CEPIA2mt*) expressing the mitochondrially targeted sensor CEPIA2mt^39^ under the neuronal promoter *HuC/elavl3* (*ELAV like neuron-specific RNA binding protein 3*) and imaged mitochondria in the optic tectum, a dorsally located part of the midbrain, where *tmbim5* transcript (Fig. 1I) and increased cell death in *tmbim5*^−/−^ larvae were detected (Fig. 1J), in *tmbim5*^−/−^;*Tg*(CEPIA2mt) and *tmbim5*^+/+^;*Tg*(*CEPIA2mt*) larvae (Fig. 3A). We quantified Ca^2+^ content by exposing immobilized living zebrafish larvae to the uncoupling agent CCCP which induces mitochondrial depolarization and, as a consequence, a release of Ca^2+^ from mitochondria. No differences between *tmbim5*^+/+^ and *tmbim5*^−/−^ larvae were observed implying that Tmbim5 deficiency in larvae had no major effect on mitochondrial Ca^2+^ levels in vivo (Fig. 3A, B). These results imply that in vivo matrix Ca^2+^ levels under conditions of physiological cytosolic Ca^2+^ levels are not affected by Tmbim5. To corroborate this, we also measured Ca^2+^ efflux upon depolarization with CCCP ex vivo in isolated mitochondria, quantifying extramitochondrial Ca^2+^ levels using a specific dye. CCCP treatment results in release of all matrix Ca^2+^ and reflects mitochondrial Ca^2+^ levels (Fig. 3C). Consistent with our in vivo measurements using CEPIA2mt, this approach revealed no differences in Ca^2+^ levels between WT and *tmbim5*^−/−^ mitochondria (Fig. 3C).

Previous reports demonstrated that TMBIM5 interacts and inhibits the m-AAA protease AFG3L2. TMBIM5 was found to be rapidly degraded in response to mitochondrial hyperpolarization resulting in activation of AFG3L2 and degradation of inner membrane and matrix proteins, including subunits of respiratory complex I^31^. Own previous work reported a reduction in complex III subunit UQCRC1 in TMBIM5 knockout human HAP1 cells^28^. We therefore also assessed the impact of Tmbim5 loss on mitochondrial function by analyzing respiratory chain activity and redox homeostasis in *tmbim5*^−/−^ larvae. Although ATP production remained unchanged (Fig. 3D), in-gel activity assays revealed a reduction in complex I activity (Fig. 3E, Supplementary Fig. 3), consistent with previous reports of diminished complex I levels caused by enhanced m-AAA protease–mediated degradation in TMBIM5-deficient HEK cells^31^. Spectrophotometric enzyme activity assays indicated a tendency towards a reduction in combined complex I + III and complex II activities (Fig. 3F), whereas reactive oxygen species (ROS) levels (Fig. 3G) remained unchanged. Notably, *tmbim5−/−* larvae showed evidence of some adaptive metabolic remodeling. The decreased CoQ10/CoQ10H₂ ratio (Fig. 3H) reflects accumulation of the reduced form of coenzyme Q, which can occur when electron flux through the respiratory chain is altered. This was accompanied by a tendency toward a lower NADP/NADPH ratio (Fig. 3I), indicating an overall shift toward a more reduced cellular redox state. Importantly, catalase activity was significantly elevated (Fig. 3J), demonstrating activation of antioxidant defense pathways. Collectively, these changes point to an adaptive increase in antioxidant capacity that appears to successfully compensate for subtle respiratory chain defects, likely explaining why ATP production (Fig. 3D) and ROS levels (Fig. 3G) remain unchanged despite the mild impairment in complex I activity.

### Tmbim5 does not function as an independent mitochondrial Ca²⁺ uptake pathway

The MCU complex is the major mitochondrial Ca^2+^ uptake mechanism^1,2^. To clarify whether Tmbim5, as overexpression results in human embryonic kidney cells suggested^29^, can act as an additional Ca^2+^ uptake mechanism besides the MCU complex or modify MCU activity, we crossed *tmbim5*^−/−^ mutants with *mcu* KO fish. We hypothesized that depletion of two potentially independent uptake mechanisms would be detrimental and/or alter mitochondrial Ca^2+^ uptake. *mcu* KO fish lack the capacity to uptake mitochondrial Ca^2+^ but have an otherwise unremarkable phenotype^11^.

**Fig. 4:**
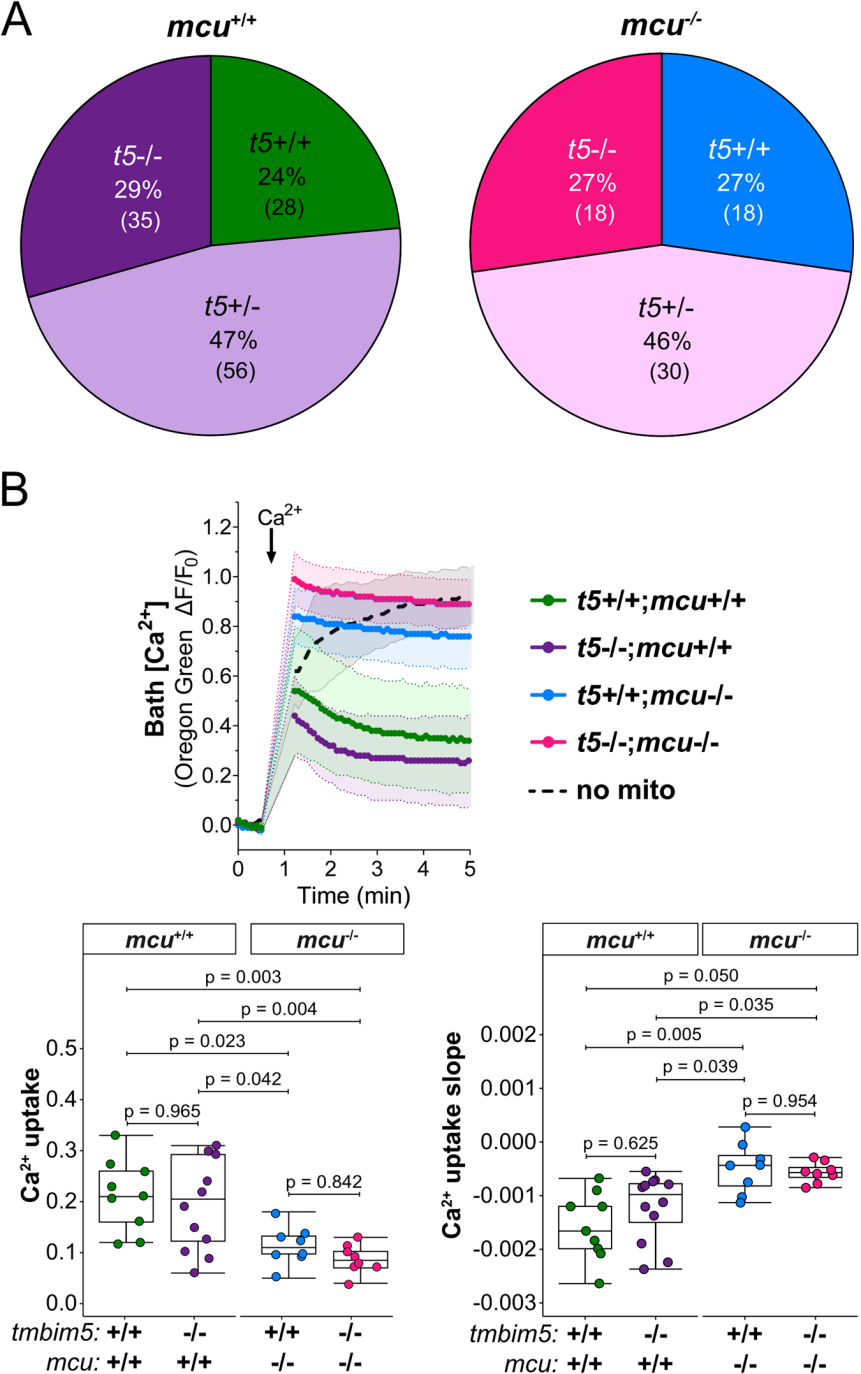
Tmbim5 does not function as an independent mitochondrial Ca²⁺ uptake pathway. **A** Genotype distribution does not deviate from the expected Mendelian ratio after *tmbim5*^+/-^*;mcu*^+/-^ zebrafish breeding. *n* is indicated in brackets. Statistical analysis: Chi-square test. **B** Loss of Tmbim5 does not significantly affect mitochondrial Ca²⁺ uptake in mitochondria isolated from *mcu*^−/−^ larvae. Mitochondria were loaded with Ca^2+^ (20 µM), revealing significantly reduced Ca^2+^ uptake in both single *mcu*^−/−^ and double *tmbim5*^−/−^*;mcu*^−/−^ knockouts. Top: fluorescence of Oregon Green 488 BAPTA-5N was normalized to the initial value (F_0_). Data are presented as mean ± SD ΔF/F_0_ traces. Gaps in the measurements are due to technical limitations of the equipment and procedure, which required pauses in fluorescence readings during reagent addition. Bottom: quantification of Ca^2+^ uptake, calculated within 2 min after CaCl_2_ addition as the difference between the maximum ΔF/F_0_ value after Ca^2+^ addition and the ΔF/F_0_ value reached 2 min later and Ca^2+^ uptake slope. Samples without mitochondria (no mito) were used as controls. Results are presented as box-and-whisker plots (box: 25th–75th percentile; whiskers: min to max), with each dot representing an independent biological replicate (*n* = 8–12). Statistical analysis: one-way ANOVA followed by post-hoc Tukey HSD test (Ca^2+^ uptake) or Kruskal-Wallis test with Dunn’s post-hoc test (slope).

We found no differences in the distribution of adult zebrafish genotypes following a heterozygous cross (Fig. 4A), suggesting no additive detrimental effect on development. We also measured mitochondrial Ca^2+^ uptake in isolated mitochondria by quantifying external Ca^2+^ levels in the bath (Fig. 4B). However, because the addition of Ca^2+^ required a ∼40 s interruption of fluorescence recording, the earliest phase of the uptake response could not be captured. We therefore report uptake slopes from the first measurable segment of the trace, which are reliable for comparisons between genotypes but not intended to represent absolute uptake rates.

These experiments confirmed the previous observation that mitochondria from Mcu-deficient zebrafish show almost no Ca^2+^ uptake^11^. There was no additional effect of *tmbim5* knockout on Ca^2+^ uptake. Together, these results imply that Tmbim5 does not constitute a relevant mitochondrial Ca^2+^ uptake system independent of Mcu. It does not rule out a modulatory effect of Tmbim5 on the MCU complex.

### Zebrafish Slc8b1 is a possible ortholog of the mitochondrial Na⁺/Ca²⁺ exchanger NCLX

TMBIM5 might also constitute an additional mitochondrial Ca^2+^ efflux system^30,31^ besides sodium-dependent mitochondrial Ca^2+^ release mediated by NCLX^17^ or TMEM65^20–23^. To clarify whether double knockout of Tmbim5 with a protein mediating mitochondrial Ca^2+^ efflux would be detrimental, we first had to generate NCLX- respectively Tmem65-deficient fish. Tmem65 knockdown by morpholinos results in an early lethality caused by impaired cardiac development^40^. Complete knockout of this protein in fish has not been described. We therefore tried to generate a knockout using CRISPR/Cas9 technology^35^ which failed despite considerable effort, thus precluding us from analyzing the effect of Tmem65 on mitochondrial Ca^2+^ efflux. Regarding NCLX, we faced the problem that the identity of NCLX in fish was unknown. We therefore focussed on a protein of 308 amino acids with the highest homology (24%) to human NCLX and a predicted sodium/calcium exchanger domain at its N-terminus (Supplementary Fig. 4A) encoded by *slc8b1*. It is noteworthy that the predicted fish Nclx protein is only half as big as human NCLX which has a length of 584 amino acids.

**Fig. 5:**
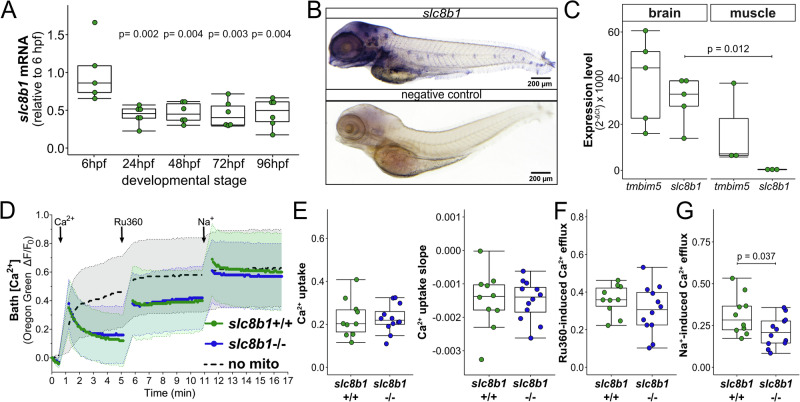
Zebrafish Slc8b1 is the likely ortholog of the mitochondrial Na⁺/Ca²⁺ exchanger NCLX. **A** *slc8b1* mRNA expression at different developmental stages, quantified using qPCR. Expression was normalized to 6 hpf, with *18S* used as a reference gene. Results are shown as box-and-whisker plots (box: 25th–75th percentile, whiskers: min–max), with each dot representing an independent biological replicate (*n* = 5–6, each RNA sample was isolated from 30 embryos/larvae). Statistical analysis: one-way ANOVA with post-hoc Tukey HSD test (*p*-values for comparisons with 6 hpf). **B** *slc8b1* transcript detected by whole-mount in situ hybridization in 4 dpf wild-type zebrafish. A sense probe was used as a negative control. Number of experiments = 3. **C** Comparative expression of *slc8b1* and *tmbim5* in the brain and skeletal muscle of adult zebrafish, assessed by qPCR. Data are presented as box-and-whisker plots, each dot representing an independent biological replicate (*n* = 3–5, each RNA sample pooled from 2 fish). Statistical analysis: *t*-test with Benjamini-Hochberg (BH) correction for multiple comparisons. **D** Functional analysis of mitochondrial Ca^2+^ transport in mitochondria isolated from *slc8b1*^−/−^ and WT larvae, using Oregon Green 488 BAPTA-5N fluorescence measurements. Changes in bath Ca^2+^ levels measured under basal conditions and after treatment with Ru360 (10 µM) to inhibit mitochondrial Ca^2+^ uptake. Upon addition of Na^+^ (10 mM), a reduced Na^+^-dependent Ca^2+^ efflux was observed in *slc8b1*^−/−^ mitochondria. Samples without mitochondria (no mito) were used as controls. Fluorescence was normalized to initial values (F_0_), and data are presented as mean ± SD ΔF/F_0_ traces. **E** *slc8b1* knockout does not affect Ca^2+^ uptake into mitochondria. Quantification of mitochondrial Ca^2+^ uptake and slope was performed 2 min after CaCl_2_ addition. **F** No significant difference in Ca^2+^ efflux following Ru360 treatment. Efflux was estimated by subtracting the last ΔF/F_0_ value after CaCl_2_ addition from the maximum ΔF/F_0_ after Ru360 addition. **G** Reduced Ca²⁺ efflux from *slc8b1*^*−/−*^ mitochondria after Na⁺ addition compared to WT. Efflux was quantified as the difference between the last ΔF/F_0_ value after Ru360 addition and the maximum ΔF/F_0_ after Na^+^ addition. **E**–**G** Results are presented as box-and-whisker plots, with each dot representing an independent biological replicate (*n* = 10–12). Statistical analysis: *t*-test.

We designed a gRNA to target the conserved exchanger domain, which led to the insertion of 17 bp, resulting in a frameshift mutation, disrupting the proposed transporter function of Slc8b1 (Supplementary Fig. 4B). This resulted in a decrease in *slc8b1* mRNA levels. There were no compensatory changes of *tmbim5* mRNA (Supplementary Fig. 4C). *slc8b1* (*nclx*) mRNA levels peaked at six h post fertilization and declined thereafter in wildtype embryos (Fig. 5A). In situ hybridization revealed that *slc8b1* is strongly expressed in the brain and in neuromasts, which are sensory patches distributed on the head and along the lateral line of fish^41^ (Fig. 5B). In adult fish, *tmbim5* and *slc8b1* are both highly expressed in the brain but *slc8b1* is expressed at a low level in skeletal muscles (Fig. 5C). There is therefore some but no complete overlap of the expression patterns of *tmbim5* and *slc8b1*.

To assess whether Slc8b1 mediates Na^+^-dependent Ca^2+^ efflux from mitochondria, we measured bath Ca^2+^ levels in mitochondria isolated from WT and *slc8b1*^−/−^ larvae. After loading mitochondria with Ca^2+^, MCU-mediated uptake was inhibited with Ru360, followed by stimulation of Na^+^-dependent Ca^2+^ efflux with a Na^+^-containing buffer (Fig. 5D). Because the addition of Ca^2+^, Ru360 and Na^+^ required pausing the recording for ~40 s, fluorescence tracking could not capture the initial portion of the efflux response. In some samples, Ca^2+^ efflux progressed rapidly during this gap, reaching near-maximal extramitochondrial Ca^2+^ levels before recordings resumed. Consequently, this assay is appropriate for comparing relative efflux capacity between genotypes, but not for determining absolute transport rates.

*slc8b1*^−/−^ mitochondria had a similar Ca^2+^ uptake (Fig. 5E) and response to Ru360 (Fig. 5F) to WT. However, sodium-dependent Ca^2+^ efflux (Fig. 5G) was significantly but not completely impaired supporting Slc8b1 as a component of this process and a possible NCLX ortholog.

### Combined loss of Tmbim5 and Slc8b1 severely disrupts mitochondrial Ca^2+^ homeostasis

**Fig. 6:**
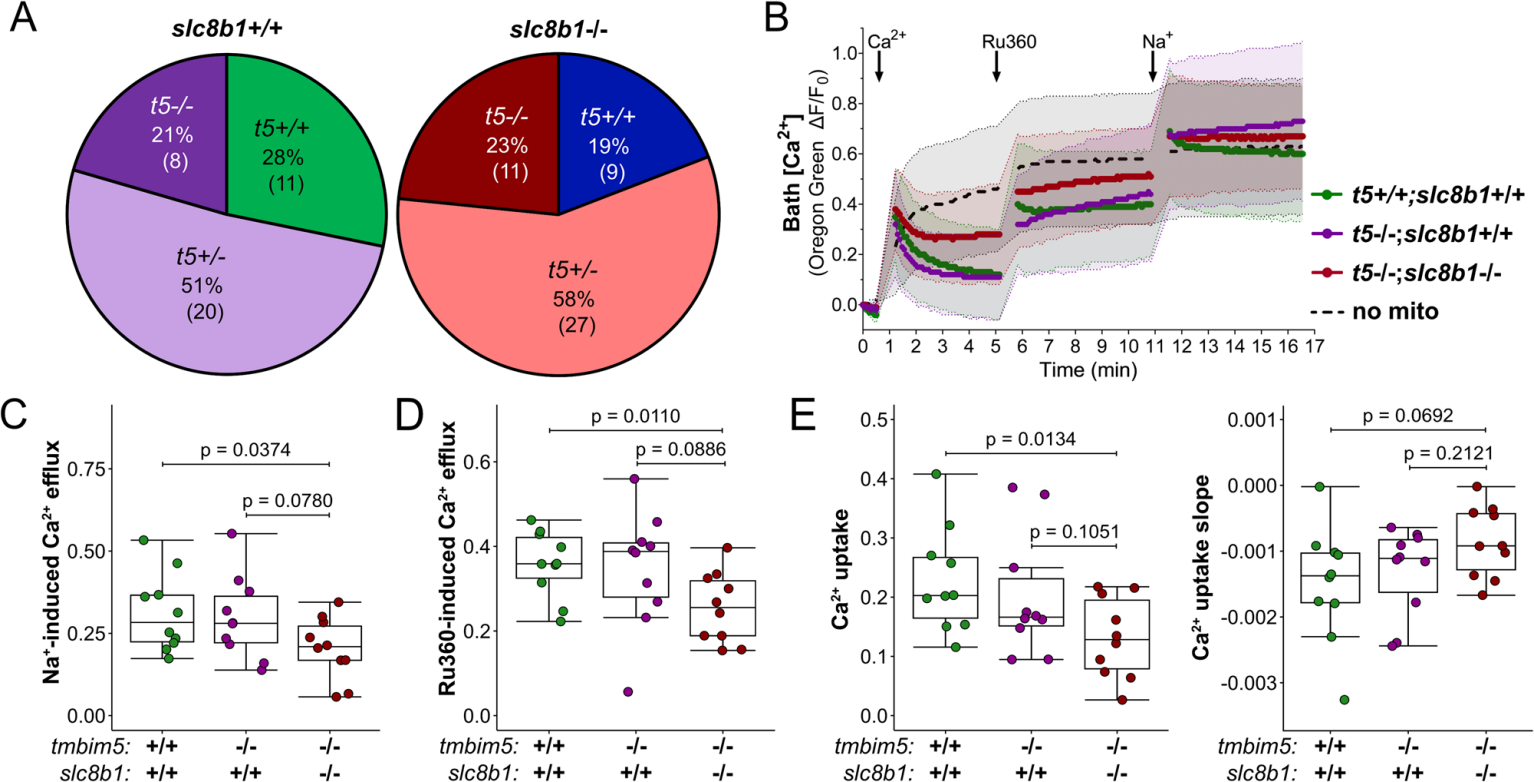
Combined loss of Tmbim5 and Slc8b1 severely disrupts mitochondrial Ca^2+^ homeostasis. **A** Genotype distribution of offspring from heterozygous *tmbim5*^+/-^*;slc8b1*^+/-^ zebrafish breeding does not differ from expected Mendelian ratios. Sample sizes are indicated in brackets. Statistical analysis: Chi-square test, number of experiments = 3. **B** Changes in bath Ca^2+^ measured by Oregon Green 488 BAPTA-5N in mitochondria isolated from wild-type (WT), *tmbim5*^−/−^, and *tmbim5*^−/−^*;slc8b1*^−/−^ larvae. Mitochondria were first loaded with Ca^2+^ (20 µM), followed by the addition of Ru360 (10 µM) to inhibit Ca^2+^ influx. Next, Na^+^ (10 mM) was added to assess Ca^2+^ efflux via NCLX activity, showing reduced Na^+^-dependent Ca^2+^ efflux in *slc8b1*^*−/−*^ mitochondria. Samples without mitochondria (no mito) were used as controls. Fluorescence was normalized to the initial value (F_0_). Data are presented as mean ± SD ΔF/F_0_ traces. **C** Reduced Na^+^-dependent Ca^2+^ efflux in mitochondria from *tmbim5*^−/−^*;slc8b1*^−/−^ larvae compared to WT. The amount of Ca^2+^ efflux following Na^+^ addition was estimated by subtracting the last ΔF/F_0_ value obtained after Ru360 addition from the maximal ΔF/F_0_ value after Na^+^ addition. **D** Decreased Ca^2+^ efflux from mitochondria isolated from *tmbim5*^*−/−*^*;slc8b1*^*−/−*^ larvae. The amount of Ca^2+^ efflux after Ru360 addition was estimated by subtracting the last ΔF/F_0_ value obtained after CaCl_2_ addition from the maximal ΔF/F_0_ value after Ru360 addition. **E** Reduced Ca^2+^ uptake and a tendency towards less negative Ca^2+^ uptake slope by mitochondria from *tmbim5*^−/−^*;slc8b1*^−/−^ larvae compared to WT. Ca^2+^ uptake was quantified 2 minutes after CaCl_2_ addition. **C**–**E** Results are presented as box-and-whisker plots (box: 25th–75th percentile, whiskers: min–max), with each dot representing an independent biological replicate (*n* = 10). Statistical analysis: two-way ANOVA followed by *t*-test or Mann–Whitney test.

Having established Slc8b1 as a relevant mitochondrial Ca^2+^ efflux system, we next examined whether removing both transport systems would be deleterious. The Mendelian distribution of offspring was as expected (Fig. 6A), indicating that the double knockout was not embryonically lethal. However, quantitative assessment of Ca^2+^ fluxes revealed significant perturbations in *tmbim5*/*slc8b1* double knockout mitochondria obtained from larvae (Fig. 6B).

Consistent with findings from mammalian cells, where combined TMBIM5 and NCLX dysfunction compromised mitochondrial Ca^2+^ handling capacity^30,31^ and impaired ruthenium red-sensitive Ca^2+^ efflux^30^, we observed similar alterations. Both Na^+^-stimulated efflux (Fig. 6C) and efflux after blocking further import with Ru360 (Fig. 6D) were reduced. Additionally, the capacity for Ca^2+^ uptake was decreased in *tmbim5*/*slc8b1* double knockout mitochondria (Fig. 6E), and at this stage, we considered a reduced electrochemical driving force as a possible explanation. The Ca^2+^ uptake slope also showed a tendency towards being less negative in the double KO, but this effect was not as significant as the effect on Ca²⁺ uptake level (Fig. 6E), suggesting that the double knockout primarily limits Ca²⁺ retention capacity rather than uptake rate. It is important to note that altered efflux could reflect two alternative scenarios: either reduced efflux because mitochondria load less Ca^2+^, or reduced uptake as a consequence of inefficient efflux leading to Ca^2+^ overload. To address this possibility, we next measured basal mitochondrial Ca^2+^ levels and mitochondrial membrane potential in vivo to gain a closer-to-physiological view of how Tmbim5 and Slc8b1 loss affect mitochondrial Ca^2+^ homeostasis and examine the organism-level consequences of the double knockout.

### Tissue-specific effects of dual Ca^2+^ transport system disruption

**Fig. 7:**
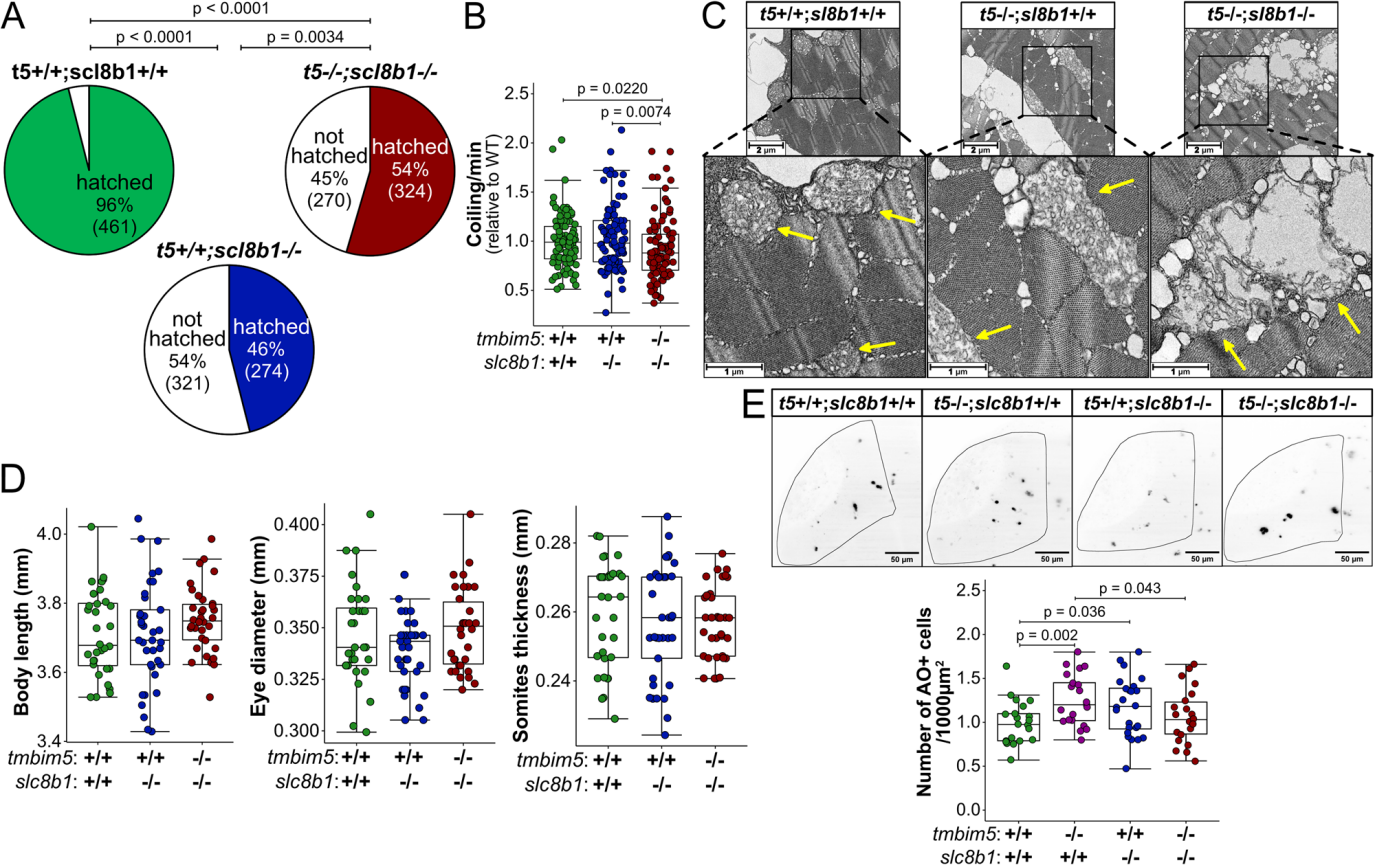
Tissue-specific effects of dual Ca^2+^ transport system disruption. **A** Reduced hatching efficiency of *slc8b1*^−/−^ and *tmbim5*^−/−^*;slc8b1*^−/−^ larvae. *n* is indicated in brackets. Number of experiments = 3. Statistical analysis: Chi-square test. **B** Decreased coiling activity of *tmbim5*^−/−^*;slc8b1*^−/−^ embryos at 1 dpf. The mean number of coiling movements per minute, normalized to WT from the same experiment, is shown as box-and-whisker plots (box: 25th–75th percentile; whiskers: min to max). Each dot represents an individual embryo (*n* = 94–96). Number of experiments = 3. Statistical analysis: two-way ANOVA followed by Mann–Whitney test. **C** Aberrant mitochondrial morphology in *tmbim5*^*−/−*^*;slc8b1*^*−/−*^ in the muscle of double knockout larvae. Representative transmission electron microscopy images of mitochondria from skeletal muscle of 5 dpf WT, *tmbim5*^*−/−*^, and *tmbim5*^*−/−*^*;slc8b1*^*−/−*^ zebrafish larvae. Arrows indicate mitochondria in magnified images. **D** Normal morphology and body size of *slc8b1*^−/−^ and *tmbim5*^−/−^*;slc8b1*^−/−^ larvae at 5 dpf. Morphometric measurements (body length, eye diameter, and somite thickness) are presented as box-and-whisker plots, with each dot representing an individual larva (*n* = 32–34). Statistical analysis: one-way ANOVA with post-hoc Tukey HSD test (body length and eye diameter) or Kruskal-Wallis test with Dunn’s post-hoc test (somite thickness). Number of experiments = 3. **E** Increased cell death in the optic tectum of *tmbim5*^−/−^ and *slc8b1*^−/−^, but not *tmbim5*^*−/−*^*;slc8b1*^−/−^ larvae analyzed with AO staining. Representative images of WT, *tmbim5*^−/−^, *slc8b1*^−/−^ and *tmbim5*^−/−^*;slc8b1*^−/−^ larvae (black dots indicate high AO signals). Quantification results are presented as box-and-whisker plots, with each dot representing an individual larva (*n* = 21–24, number of experiments = 3). Statistical analysis: two-way ANOVA followed by *t*-test.

We next investigated the effect of double knockout on the phenotypes associated with Tmbim5 loss of function to understand how disruption of multiple Ca^2+^ transport pathways affects different tissues. Similarly to *tmbim5*^−/−^ larvae (Fig. 1E), *slc8b1*^−/−^ and *tmbim5*/*slc8b1* KO larvae exhibited a lower hatching efficiency compared to WT (Fig. 7A). Also, coiling activity, which precedes hatching, was reduced in *tmbim5*/*slc8b1* embryos compared to wildtype and *slc8b1*^−/−^ (Fig. 7B), suggesting skeletal muscle impairment. This correlated with pronounced alterations in mitochondrial ultrastructure in double knockout animals shown by transmission electron microscopy (Fig. 7C). Despite these changes, spontaneous (Supplementary Fig. 5A) and light-induced (Supplementary Fig. 5B) locomotor activity of *slc8b1*^−/−^ and *tmbim5*/*slc8b1* double knockout larvae were normal.

Remarkably, all phenotypes associated with reduced size i.e. larval body length, eye diameter and somite thickness were surprisingly similar between wildtype, *slc8b1*^−/−^ and *tmbim5*/*slc8b1* double knockout larvae (Fig. 7D). Double knockout also rescued the increased cell death in the central nervous system observed in *tmbim5*^−/−^ and in *slc8b1*^−/−^ larvae (Fig. 7E). Together these data imply that concurrent loss of both transport systems affects different tissues differentially. While the effects on coiling were more pronounced, corresponding to severe disruption of the mitochondrial cristae architecture, the effects of *tmbim5* loss of function on body size and increased cell death in nervous tissue were rescued by concomitant knockout of *tmbim5* and *slc8b1*. This suggests tissue-specificity of mitochondrial Ca^2+^ transport systems or tissue-specific additional function of Tmbim5 or Slc8b1.

### Mitochondrial Ca^2+^ and membrane potential changes reveal tissue-specific compensation mechanisms

To address this tissue specificity, we therefore decided to assess changes in steady-state mitochondrial Ca^2+^ and membrane potential levels of WT, *tmbim5*^−/−^ and *tmbim5*/*slc8b1* KO larvae in susceptible and non-susceptible tissues, here brain and muscle. To quantify basal mitochondrial Ca^2+^ levels, we injected plasmid DNA encoding the 4mtD3cpv Ca^2+^ probe into zebrafish embryos^42^. This FRET-based sensor allows ratiometric imaging and quantification of Ca^2+^ amounts independent of probe expression level^43,44^. To quantify mitochondrial membrane potential, we injected tetramethylrhodamine ethyl ester (TMRE) into the yolk of 1-cell stage zebrafish embryos as described in Vicente et al.^45^. TMRE is a positively charged dye known to accumulate in mitochondria due to their membrane potential. Two days after injection, we recorded a TMRE signal in brain and skeletal muscle that was significantly reduced after depolarization with carbonyl cyanide 3-chlorophenylhydrazone (CCCP) used as positive control.

**Fig. 8:**
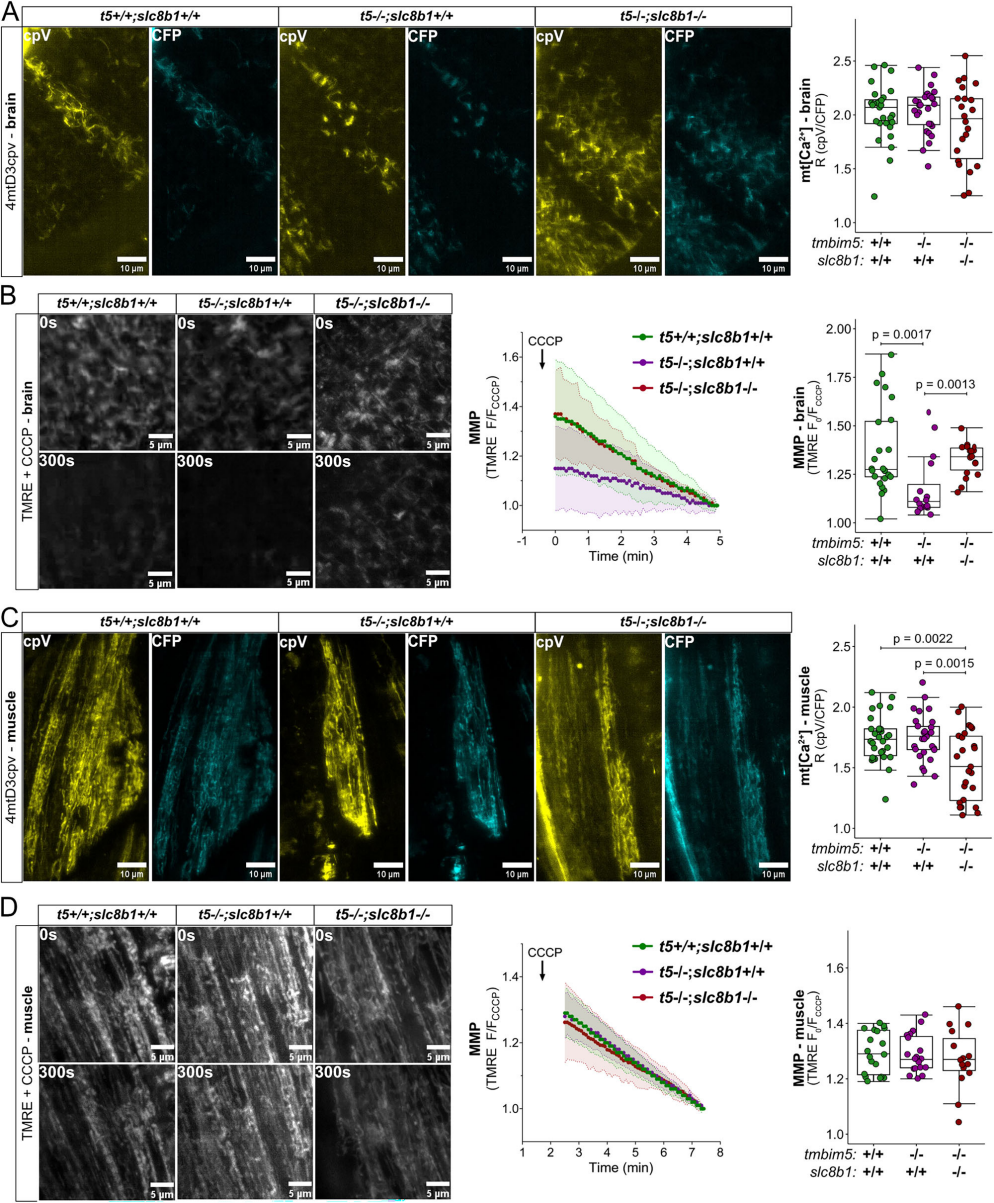
Mitochondrial Ca²⁺ and membrane potential changes reveal tissue-specific compensation mechanisms. **A** Basal mitochondrial Ca^2+^ level in the brain is not affected by knockout of *tmbim5* or *tmbim5*/*slc8b1* double knockout larvae. Left: representative images of brain (optic tectum) of 2 dpf larvae with transient and mosaic expression of the 4mtD3cpv Ca^2+^ probe. Right: mitochondrial [Ca^2+^], assessed from the ratio of cpV to CFP (R), is shown as a box-and-whisker plot (box: 25th-75th percentile; whiskers: min to max), with each point representing an individual larva (*n* = 22-29). Statistical analysis: two-way ANOVA followed by *t*-test. **B** Decreased mitochondrial membrane potential in *tmbim5*^−/−^, but not in *tmbim5*^−/−^*;slc8b1*^−/−^ zebrafish observed in vivo in the optic tectum of 2 dpf larvae. Zebrafish embryos were injected with TMRE (1 nl of 2 mM) at the 1-cell stage and imaged in vivo using LSFM at 2 dpf. CCCP (10 µM) was used to depolarize mitochondria, and the F_0_/F_CCCP_ ratio was calculated. Left: representative images of the ROI in the brain at the beginning and after 5 min of CCCP treatment. Right: changes in membrane potential (MMP) measured in the brain by TMRE. Data are presented as mean ± SD F/F_CCCP_. MMP, calculated as the F_0_/F_CCCP_ ratio, is plotted as box-and-whisker plots, with each dot representing an individual larva (*n* = 16–24). Statistical analysis: two-way ANOVA followed by Mann–Whitney test. **C** Decreased basal mitochondrial Ca^2+^ level in muscles of *tmbim5*^−/−^;*slc8b1*^−/−^ larvae. Left: representative images of muscle of 2 dpf larvae with transient and mosaic expression of the 4mtD3cpv Ca^2+^ probe. Right: mitochondrial [Ca^2+^], assessed as in (**A**), is shown as a box-and-whisker plot, with each point representing an individual larva (*n* = 25–30). Statistical analysis: two-way ANOVA followed by t-test. **D** Unaffected mitochondrial membrane potential in *tmbim5*^−/−^ and in *tmbim5*^−/−^;*slc8b1*^−/−^ zebrafish measured in vivo in the muscle of 2 dpf larvae. Measurements were performed as described in (**B**). Left: representative images of the ROI in the muscle at the beginning and after 5 min of CCCP treatment. Right: changes in MMP measured in the muscle by TMRE. Data are presented as mean ± SD F/F_CCCP_. MMP, calculated as the F_0_/F_CCCP_ ratio, is plotted as box-and-whisker plots, with each dot representing an individual larva (*n* = 15–20). Statistical analysis: two-way ANOVA followed by Mann–Whitney test.

In the brain, we did not observe any significant changes in basal mitochondrial Ca^2+^ levels of *tmbim5*^−/−^ or *tmbim5*/*slc8b1* double knockout larvae (Fig. 8A). However, mitochondrial membrane potential was reduced in Tmbim5-deficient larvae and rescued in *tmbim5*/*slc8b1* double knockout (Fig. 8B). In the muscles of *tmbim5*/*slc8b1* larvae, in contrast, the mitochondrial Ca^2+^ content was significantly lower than in WT or *tmbim5* single knockout (Fig. 8C) in line with the reduced mitochondrial Ca^2+^ uptake observed in purified mitochondria (Fig. 6E). MMP was similar between the genotypes (Fig. 8D) making a reduced driving force as the cause of diminished Ca^2+^ uptake unlikely. These results, therefore, corroborate our phenotypic analysis where double knockout of *tmbim5* and *slc8b1* aggravates or ameliorates Tmbim5 loss of function in a tissue-specific manner. The beneficial effect in the brain appears not to be mediated by changes in mitochondrial Ca^2+^ homeostasis but rather via effects on the activity of the electron transfer system possibly by changes of AFG3L2-mediated remodeling of the respiratory complexes. In muscle, the opposite appears to be true.

Our results are therefore consistent with TMBIM5 functioning as a mitochondrial Ca^2+^ efflux pathway, as evidenced by the impaired Ca^2+^ handling capacity when both Tmbim5 and Slc8b1 are absent. The dysregulated mitochondrial Ca^2+^ homeostasis resulting from simultaneous disruption of these efflux pathways may directly contribute to the observed ultrastructural abnormalities. The disconnect between severe subcellular abnormalities and mild organismal phenotypes underscores the remarkable adaptability of mitochondrial Ca^2+^ homeostatic systems in vivo.

### Transcriptional changes of genes encoding the mitochondrial Ca^2+^ machinery differ between brain and skeletal muscle

To investigate whether the differential effects of Tmbim5 and Slc8b1 loss in brain and muscle result from compensatory transcriptional changes, we analyzed the expression levels of genes involved in mitochondrial Ca^2+^ handling: *mcu*, *micu1–3*, *efhd1*, *letm1*, *letm2*, *letmd1*, *slc8b1*, *tmem65*, and *afg3l2*. *slc25a28*, encoding a Ca^2+^-independent iron transporter, served as control. We compared expression between wildtype and *tmbim5* KO, wildtype and *tmbim5/slc8b1* double knockout, and *tmbim5* KO versus double knockout in both tissues.

**Fig. 9:**
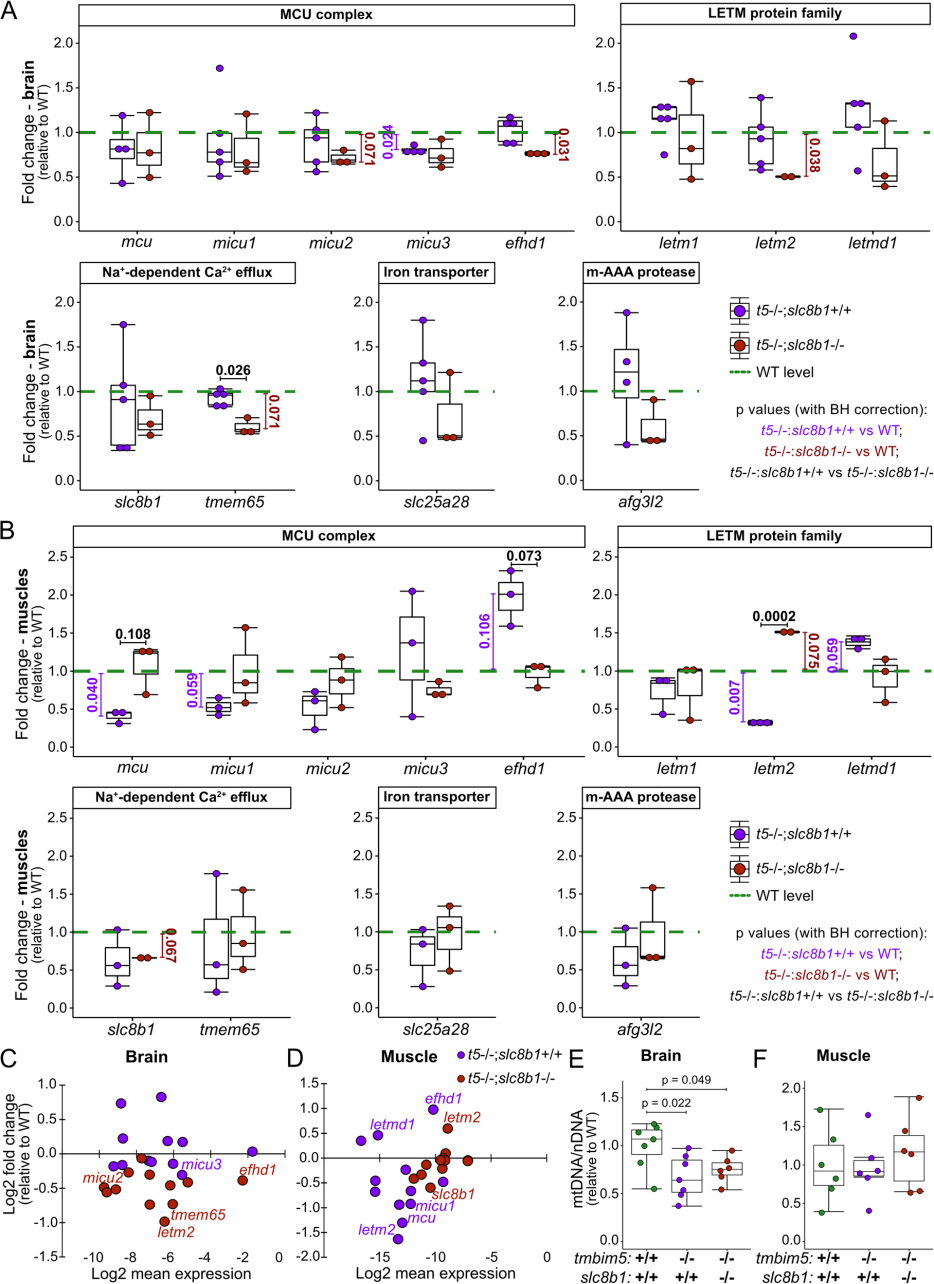
Transcriptional changes of genes encoding the mitochondrial Ca^2+^ machinery differ between brain and skeletal muscle. **A** *micu3* is downregulated in the brain of *tmbim5*^−/−^ fish, while *efhd1* and *letm2* expression is reduced in the brain of *tmbim5*^−/−^;*slc8b1*^−/−^. **B** In the muscle of *tmbim5*^−/−^ fish, *mcu* and *letm2* are downregulated; however, in *tmbim5*^−/−^;*slc8b1*^−/−^ fish, *letm2* is upregulated in the muscle. qPCR results presented as MA plots showing the relation between fold change and the mean expression levels of examined genes in the brain (**C**) and muscle (**D**) calculated as 2^(-ΔCq)^. **A**–**D** mRNA levels of proteins involved in mitochondrial Ca^2+^ transport were quantified by qPCR in the brain and skeletal muscles of adult (8–14-month-old) zebrafish and normalized to WT. *rpl13a* and *ef1a* were used as reference genes. Results are presented as box-and-whisker plots (box: 25th–75th percentile; whiskers: min to max), with each dot representing an independent biological replicate (*n* = 2–6, with each RNA sample isolated from 2 fish). Number of experiments = 3–6. Statistical analysis: one-sample *t*-test (comparisons with WT) or *t*-test with BH correction for multiple comparisons. *p*-values shown below the boxplots indicate comparisons with WT: magenta values represent comparisons with *tmbim5* knockout, and red values represent comparisons with *tmbim5/slc8b1* double knockout. Black *p*-values above boxplots indicate comparisons between *tmbim5* knockout and *tmbim5/slc8b1* double knockout. **E** The mitochondrial (mtDNA) to nuclear DNA (nDNA) ratio is decreased in the brain of *tmbim5*^−/−^ and *tmbim5*^−/−^;*slc8b1*^−/−^ zebrafish. **F** Unchanged mtDNA to nDNA ratio in the skeletal muscle of *tmbim5*^−/−^ and *tmbim5*^−/−^;*slc8b1*^−/−^ zebrafish. **E**, **F** The mtDNA/nDNA ratio was quantified by qPCR and normalized to WT. *mt-nd1* (*mitochondrial NADH dehydrogenase 1*) was used as a marker for mtDNA, while *ef1a* was used for nDNA. Results are presented as box-and-whisker plots, with each dot representing an independent biological replicate (*n* = 6). Number of experiments = 2. Statistical analysis: two-way ANOVA followed by *t* test.

In the brain, *micu3* was significantly downregulated in *tmbim5* KO (Fig. 9A). Double knockout (*tmbim5/slc8b1*) resulted in downregulated *efhd1* and *letm2* levels compared to wild-type, while *tmem65* was specifically reduced compared to *tmbim5* KO (Fig. 9A). In skeletal muscle, *tmbim5* KO reduced *mcu* and *letm2* while increasing *efhd1* (Fig. 9B). Conversely, double knockout showed *letm2* upregulation compared to both wild-type and *tmbim5* KO (Fig. 9B). Notably, the expression levels of all mitochondrial calcium machinery genes were broadly reduced in double knockout brain tissue (two-way ANOVA: *p* = 0.000002, Fig. 9C) and in *tmbim5* KO muscle, although less evidently (*p* = 0.01816, Fig. 9D). These changes corresponded to a significant reduction in mitochondrial DNA content in the brains of Tmbim5-depleted fish that persisted in the *tmbim5*/*slc8b1* double KO (Fig. 9E) but not in muscle (Fig. 9F).

The downregulation of *micu3* in brain and *mcu* in muscle as well as the upregulation of *efhd1*, which is an inhibitor of the MCU^46^, is in line with compensatory changes reducing mitochondrial Ca^2+^ influx suggesting that Tmbim5 is mainly part of an efflux system. We posit that *letm2* may mediate tissue-specific effects, being downregulated in protective brain but upregulated in affected muscle. While previously being reported as testis-specific^47^, *letm2* is expressed in the brain (Fig. 9B) and even dominates over *letm1* in zebrafish muscle (Fig. 9D). Not much is known about its function. Alternatively, coordinated downregulation of the entire mitochondrial Ca^2+^ machinery may protect brain tissue against *tmbim5* deficiency by shifting energy supply to glycolysis as described for astrocyte-specific *Nclx* knockout in mice^48^.

## Discussion

Our findings establish Tmbim5 as critical for growth and muscle function in zebrafish and demonstrate that it functions predominantly as a mitochondrial Ca^2+^ efflux rather than influx transport system. This conclusion is based on the lack of additive effects in *tmbim5*/*mcu* double knockouts and the functional impairment when both efflux pathways (*tmbim5* and Na^+^-dependent Ca^2+^ efflux via *slc8b1*) were disrupted simultaneously. Surprisingly, *slc8b1* knockout alone produced minimal phenotypic effects, contrasting with the embryonic lethality observed in mammalian *NCLX* knockouts. Even more unexpected, the double knockout of both efflux pathways was protective in some tissues but detrimental in others. These results suggest that the functions of Tmbim5 and Slc8b1 can be tissue-specific and indicate the existence of additional, yet uncharacterized mitochondrial Ca^2+^ transport mechanisms.

Tmbim5 deficiency decreased the size of zebrafish larvae and weight of adults in line with knockdown of the *tmbim5* ortholog in snails^49^ but not in mice^29^. However, similar to *Tmbim5* mutant mice that suffer from myopathy, myofiber cross-sections were smaller in *tmbim5*^−/−^ fish, indicating muscle atrophy. This was particularly pronounced in slow-twitch muscles, which contain abundant mitochondria and rely heavily on oxidative phosphorylation compared to fast-twitch fibers^38^, suggesting a link with compromised mitochondrial function. These pathological alterations of muscle function probably underlie the delayed escape of Tmbim5-deficient embryos from the chorion which is mediated by tail coiling^36^. Interestingly, *Mcu* knockout in mice also negatively affects size^8^ and affects proper skeletal muscle trophism^50,51^. MICU1-deficient mice also showed severe growth defects^52^ and marked muscle weakness, easy fatigue and smaller muscle fibers^52,53^. In addition, loss-of-function mutations in human *MICU1* are associated with myopathy^54–56^. Together, these observations corroborate the role of Tmbim5 in the regulation of growth and muscle function and point out similarities between the effects of Tmbim5, Mcu and Micu1 loss.

Two recent studies demonstrated that TMBIM5 functions as a pH-dependent mitochondrial Ca^2+^ efflux transporter^30,31^. Zhang et al., in contrast, found that TMBIM5 overexpression in HEK cells enhanced mitochondrial Ca^2+^ uptake following ER Ca^2+^ release^29^, suggesting that TMBIM5 may operate bidirectionally under certain conditions. These discrepancies may partly reflect the intrinsic differences between overexpression and loss-of-function approaches, as overexpression can produce non-physiological effects, whereas TMBIM5 knockout in HEK cells, as also reported by Zhang et al., shows no significant alteration of Ca²⁺ uptake^29^, consistent with the efflux role proposed by other groups^30,31^. To clarify the physiological relevance of this, we now generated two double knockout zebrafish lines: one lacking both Tmbim5 and Mcu, the primary mitochondrial Ca^2+^ influx mediator, and another deficient in Tmbim5 and Slc8b1, the zebrafish ortholog of NCLX, the presumed major mitochondrial Ca^2+^ efflux pathway. Surprisingly, both double knockout lines were viable and exhibited no overt organismal phenotypic alterations.

The absence of enhanced mitochondrial Ca^2+^ uptake deficits in *tmbim5*/*mcu* double knockout zebrafish argues against Tmbim5 functioning as a major Ca^2+^ influx channel as this would have likely caused a more severe phenotype and would have reduced Ca^2+^ uptake independently of Mcu function. Two alternative mechanisms remain plausible to reconcile these data with previous reports: First, Tmbim5 may facilitate Ca^2+^ transport specifically at high Ca^2+^ concentrations. The increased uptake observed in TMBIM5-overexpressing cells after ATP stimulation^29^ might be relevant at mitochondria-ER contact sites, where local Ca^2+^ reaches up to 100 μM–conditions not tested in our system. Second, Tmbim5 may function as a regulatory modifier of MCU complex activity rather than as an autonomous Ca^2+^ influx channel. This hypothesis is supported by recent proteomic analyses identifying Tmbim5 as a component of the MCU interactome^46,57^. The normal mitochondrial Ca^2+^ uptake observed in Tmbim5-deficient mitochondria also argues against significant alteration of MCU complex function through the proposed AFG3L2-EMRE regulatory axis^58,59^. A reduction in Ca^2+^ uptake was observed only in mitochondria lacking both Tmbim5 and Slc8b1. However, these mitochondria also exhibited decreased Na^+^-dependent and Na^+^-independent Ca^2+^ efflux. An important limitation when interpreting the mitochondrial efflux assays is that apparent efflux capacity inherently depends on the amount of Ca^2+^ loaded. Thus, the reduced efflux observed in double knockouts could reflect two alternative scenarios: either lower efflux simply due to reduced Ca^2+^ loading, or decreased uptake as a secondary consequence of inefficient efflux leading to Ca^2+^ overload. To address this, we measured basal mitochondrial Ca^2+^ levels in vivo, providing a more physiological context for evaluating the impact of Tmbim5 and Slc8b1 on Ca²⁺ homeostasis. In contrast, while the reduced basal Ca²⁺ levels in *tmbim5*/*slc8b1* muscle could indicate decreased Ca^2+^ influx, we interpret this as evidence of severely compromised mitochondrial integrity. Mitochondrial Ca²⁺ homeostasis is intimately linked to cristae architecture and overall organelle integrity^60–62^. Both Ca²⁺ overload and impaired Ca²⁺ fluxes can destabilize cristae junctions and may trigger mitochondrial swelling, ultimately lowering mitochondrial capacity to retain Ca²⁺. Indeed, TMBIM5^27,29,30^ and NCLX^18,63,64^ have been associated with preservation of cristae integrity, and loss of either protein in various models produces cristae disorganization and increased susceptibility to stress. Similar morphological abnormalities have been described also for loss of LETM1^16,65^, and MICU1^42,66^, each of which has been associated with swelling or cristae disorganization in different models. Our ultrastructural analysis revealed comparable abnormalities in the double *tmbim5*/*slc8b1* knockouts, supporting the notion that disrupted Ca²⁺ homeostasis contributes to structural defects, which in turn compromise the ability of mitochondria to maintain normal Ca²⁺ content. Thus, the reduced Ca²⁺ levels might reflect a secondary consequence of impaired mitochondrial integrity rather than a direct increase in efflux activity.

Our study provides the first comprehensive characterization of zebrafish *slc8b1*, establishing its expression pattern, functional properties, and loss-of-function phenotype as the probable NCLX ortholog in this model organism. Similar to murine NCLX^18^, zebrafish *slc8b1* demonstrates predominant expression in neural tissues. Functional analyses confirmed that Slc8b1 depletion significantly attenuated Na^+^-dependent mitochondrial Ca^2+^ efflux but residual Na^+^-dependent efflux persisted, suggesting potential compensatory mechanisms or alternative transport pathways, possibly Tmem65^20–23^. Interestingly, in contrast to *slc8b1*, depletion of fish *tmem65* results in lethality^40^. The structural divergence between zebrafish Slc8b1 and its mammalian counterparts–with fish Slc8b1 being approximately half the size of human NCLX and encoding only five transmembrane domains instead of twelve – also raises intriguing mechanistic questions. It is possible that zebrafish Slc8b1 may function as a homodimer, as it was observed for mammalian NCLX^67^, or potentially interact with additional protein partners to constitute a fully functional NCLX complex^67^. The milder phenotype of *slc8b1* knockout in zebrafish compared to mammalian NCLX knockout may reflect this pronounced structural divergence, which may alter its transport capacity or necessitate interaction with additional proteins. Thus, loss of Slc8b1 in zebrafish may not fully recapitulate the consequences of NCLX deletion in mammals.

Notably, the effects of the loss of Slc8b1 in *tmbim5* knockout fish differed depending on the tissue. Also in mammals, *Nclx* knockout in cardiac tissue causes lethal mitochondrial calcium overload and depolarization^18^. In neurons, *Nclx* knockout leads to mitochondrial calcium overload, which disrupts synaptic calcium transients and impairs synaptic plasticity, affecting learning and memory^48,68,69^. In astrocytes, however, *Nclx* knockout increases glycolytic flux and lactate secretion, leading to improved cognitive performance^48^. Overall, even the transport function of Nclx is not completely understood as *Slc8b1* is highly expressed in mouse liver tissue, where Na^+^-dependent Ca^2+^ efflux is minimal^70^. The arrival of TMEM65 as an additional transport system or NCLX regulator or interacting partner^20–23^ will hopefully bring some more clarity. However, recent findings highlight that the molecular identity and mechanism of mitochondrial Ca²⁺ efflux remain incompletely understood. While NCLX has long been considered the main Na⁺-dependent Ca²⁺ efflux transporter^17,71^, the latest studies have questioned this view. TMEM65 has been proposed either as an interacting partner of NCLX^23^ or as an independent Na⁺-dependent Ca²⁺ transporter^22^, and a recent structural analysis of mammalian NCLX found no Na⁺ binding site, suggesting instead a pH-dependent mechanism^19^. This observation gives rise to the possibility that NCLX and Tmbim5 collaborate with each other in Ca^2+^/H^+^ exchange which we could unfortunately not address as we did not succeed in generating Tmem65-deficient fish. This inability could be cautiously interpreted as a hint towards the importance of the transport system in zebrafish mitochondrial Ca^2+^ dynamics.

The viability of *tmbim5/slc8b1* double knockout fish, despite altered mitochondrial Ca²⁺ handling, suggests the presence of robust compensatory mechanisms or alternative pathways that maintain Ca²⁺ homeostasis when both major efflux systems are disrupted. LETM1 represents a prime candidate for such compensation. LETM1 has been proposed to mediate mitochondrial Ca²⁺/H⁺ exchange^15^, though it was originally identified as a component of the mitochondrial K⁺/H⁺ exchanger^16^. While LETM1-containing liposomes demonstrated Ca²⁺ transport capacity^15,72,73^, its physiological role as a direct Ca²⁺ transporter remains disputed. Interestingly, LETM1 might interact with TMBIM5^30^, suggesting potential functional crosstalk between these proteins. In zebrafish, *letm1* deficiency causes embryonic lethality in most but not all mutants, suggesting variable compensatory capacity^74^. However, the specific role of Letm1 in Ca²⁺ homeostasis has not been functionally characterized in this model.

Intriguingly, our gene expression analysis revealed tissue-specific regulation of *letm2*, a LETM1 paralog, rather than *letm1* itself. In *tmbim5/slc8b1* double knockouts, *letm2* was downregulated in brain tissue (where phenotypes were ameliorated) but upregulated in muscle tissue (where dysfunction was exacerbated). If Letm2 were functioning compensatorily, one would expect the opposite pattern. Unlike LETM1, LETM2 lacks Ca²⁺-binding EF-hand domains and cannot functionally replace LETM1, as evidenced by its inability to rescue LETM1 knockdown-induced mitochondrial swelling^47^). One very speculative explanation could be that LETM2 might function as a negative regulator of LETM1 activity, analogous to how MCUb inhibits MCU-mediated Ca²⁺ uptake^75^, though this hypothesis requires experimental validation and remains highly tentative given the limited functional data available for LETM2 (and the limited understanding of Letm1 in zebrafish) and should be addressed in future studies. It is likely that the tissue-specific phenotypes we observed result from a combination of reduced efflux capacity and compensatory mechanisms, as supported by the transcriptional changes detected in multiple Ca^2+^ transport and regulatory genes such as *letm2*, but also *mcu*, *micu* family members, and *efhd1*. Alternatively, the reduced mitochondrial mass might trigger a beneficial shift in energy metabolism towards glycolysis, similar to the beneficial metabolic adaptation observed in astrocyte-specific *Nclx* knockout mice^48^.

In conclusion, loss of Tmbim5 impairs zebrafish growth, muscle function and increases cell death in the brain and results in abnormal mitochondrial function as evidenced by reduced mitochondrial membrane potential and respiratory chain activity but no clear disruption of mitochondrial Ca^2+^ handling. The absence of Tmbim5 did also not exacerbate the phenotype of either *mcu* or *slc8b1* knockout zebrafish, despite some functional and morphological changes in *tmbim5*/*slc8b1* double knockout fish. Importantly, the effects of Slc8b1 loss in Tmbim5 knockout zebrafish were tissue-specific and could either restore or exacerbate the Tmbim5 absence phenotype. Although complementary cell-based studies may in the future help dissect the precise molecular mechanisms underlying the observed phenotypes, our work established tissue-specific and whole-organism consequences and crosstalk between different Ca^2+^ transport systems.

## Methods

### Animal maintenance

*tmbim5*^*+/+*^*, tmbim5*^*−/−*^, *tmbim5*^+/+^;*Tg*(*HuC:CEPIA2mt*), *tmbim5*^−/−^;*Tg*(*HuC:CEPIA2mt*), *tmbim5*^+/+^;*mcu*^−/−^, *tmbim5*^−/−^;*mcu*^−/−^, *tmbim5*^+/+^;*slc8b1*^−/−^ and *tmbim5*^−/−^;*slc8b1*^−/−^ zebrafish were used in the study. All animals were maintained following previously described methods^76^. in the Zebrafish Core Facility, a licensed breeding and research facility (PL14656251, registry of the District Veterinary Inspectorate in Warsaw; 064 and 051, registry of the Ministry of Science and Higher Education) at the International Institute of Molecular and Cell Biology in Warsaw. Adult zebrafish and larvae were kept in E3 medium (2.48 mM NaCl, 0.09 mM KCl, 0.164 mM CaCl_2_·2H_2_O, and 0.428 mM MgCl_2_·6H_2_O) at 28.5 °C. Larvae were kept in a Petri dishes (~50 larvae/dish) in an incubator under a 14 h/10 h light/dark cycle. The stages of fish development were defined as h postfertilization (hpf) and days postfertilization (dpf). Zebrafish embryos intended for stainings or in vivo imaging experiments were treated with N-phenylthiourea (PTU, Alfa Aesar Cat# 103-85-5) at final concentration of 0.003% to avoid pigmentation.

This study follows ARRIVE guidelines^77^. We have complied with all relevant ethical regulations for animal use. All experimental procedures were approved by the Local Ethical Committee for Experiments on Animals in Warsaw (permission no. WAW2/050/2022) and were performed in accordance with European and Polish regulations on animal welfare.

### Mutant and transgenic zebrafish lines

The generation of *tmbim5*^−/−^ zebrafish using CRISPR-Cas9 gene editing was previously described in Gasanov^35^. Briefly, a mix of two gRNA: 5’-GGCTGGTATGTGGGAGTCTA-3’ and 5’-CGCGGGCAGTGTGGGCCTGA-3’ (0.04 mg/ml of each) and Cas9 protein (0.2 mg/ml) was injected into the cytoplasm of one-cell stage wild-type AB zebrafish embryos.

Fish were raised after injections up to 4-months-old and were fin-clipped under anesthesia with 140 mg/ml Tricaine (MS-222, Sigma, Cat# A-5040) for DNA sampling and high resolution melting (HRM) analysis. Targeted region in the 4^th^ exon of *tmbim5* was amplified using Precision Melt Supermix (BioRad, Cat# 1725112), primers: *tmbim5 HRM forward*: 5’-CGATCTGGCCGCAGTACG-3’, *tmbim5 HRM reverse*: 5’-CCAGCCAGGAGTTGCTC-3’ and the CFX Connect RT-PCR Detection System (BioRad). Melting curve analysis was performed using Precision Melt Analysis (Bio-Rad) software. The mutation-positive ones were analyzed by DNA sequencing. Fish with the deletion of 29 nucleotides and premature STOP codon was selected as F0 founder. F0 fish was outcrossed with the AB zebrafish line, and their mutation-carrying offspring were in-crossed to generate homozygous mutants. Fish were screened via PCR using the same primers as for HRM analysis, followed by Sanger sequencing.

Genotyping of subsequent generations of *tmbim5*^+/+^, *tmbim5*^+/−^ and *tmbim5*^−/−^ fish was based on the difference in the size of PCR product that was detected by electrophoresis in 2% agarose.

For in vivo mitochondrial Ca^2+^ imaging experiments *tmbim5*^−/−^ fish were outcrossed with *Tg*(*HuC:CEPIA2mt*) zebrafish (obtained as a kind gift from prof. Jacek Kuźnicki laboratory). Their offspring were sorted for CEPIA2mt signal in the brain under the fluoroscope between 2 to 4 dpf.

To generate double knockout of *tmbim5* and *mcu*, *tmbim5*^−/−^ zebrafish were outcrossed with *mcu*^−/−^ zebrafish (obtained as a kind gift from prof. Jacek Kuźnicki laboratory). Obtained heterozygous fish were incrossed and their offspring were genotyped for *tmbim5* as described above and for *mcu* as described in Soman et al.^11^.

To generate zebrafish knockout of *nclx* the exon 5 of *slc8b1* gene was targeted with gRNA designed with free web tools: CHOPCHOP^78^ and CRISPRscan^79^, according to the protocol described in Wiweger et al.^80^. Sequences that were common in both of the predictions and were scored as low risk for off-targets were chosen. The correctness of the sequence in the targeted area in the AB zebrafish was confirmed by Sanger sequencing. The gene-specific oligonucleotide with T7 overlaps: 5’-taatacgactcactataGGAGTGACGTTCCTGGCTCTgttttagagctagaa-3’. During gRNA preparation, annealing and filling in steps were combined, and the template was prepared by PCR using BioMix Red (BioLine, Cat# BIO-25006), gene-specific and constant oligo: 5’-AAAAGCACCGACTCGGTGCCACTTTTTCAAGTTGAT AACGGA CTAGCCTTATTTTAACTTGCTATTTCTAGCTCTAAAAC-3’ (10 µM). The PCR conditions were the following: pre-incubation at 95 °C for 3 min, followed by 35 cycles of 95 °C for 15 s, 40°C for 15 s, and 68 °C for 15 s. sgRNA was prepared using 150 ng of purified PCR product and MegaScript T7 Transcription Kit (Invitrogen, Cat# A57622) according to the manufacturer’s recommendations. Thereafter, RNA was precipitated by overnight incubation with ammonium acetate at −20 °C.

gRNA was suspended in water, and the concentration was adjusted to 500 ng/μl. Cas9 protein (14 mg/ml stock, made in-house) was diluted in KCl/HEPES (200 mM/10 mM, pH 7.5) buffer to a final concentration of 600 ng/μl. The injection mixture was assembled fresh by mixing 2 μl of gRNA (1 μg), 2 μl of Cas9 (1.2 μg), and 0.5 μl phenol red (Sigma, Cat# P0290) and left for complex formation at room temperature for 5–10 min. Thereafter, the samples were kept on ice. Microneedles were pulled from borosilicate glass capillaries (Sutter; Cat# B100-75-10) using a P-1000 Flaming/Brown micropipette puller (Sutter). One nanoliter of the gRNA/Cas9/phenol red mixture was injected into the yolk at 1–2 cell-stage *tmbim5*^−/−^ zebrafish embryos using a FemtoJet microinjector (Eppendorf).

Fish were grown up after injections up to 4-months-old and were fin-clipped under anesthesia with 0.14 mg/ml Tricaine for DNA sampling and high resolution melting (HRM) analysis. Targeted region in the 5th exon of *scl8b1* was amplified using Precision Melt Supermix (BioRad, Cat# 1725112), primers: *slc8b1 HRM forward*: 5’-GTGTACCATGTGGATGTGTGTTG-3’, *slc8b1 HRM reverse*: 5’-CAGACCAGCAGTTTGAGGGTG-3’ and the CFX Connect RT-PCR Detection System (BioRad). Melting curve analysis was performed using Precision Melt Analysis (Bio-Rad) software. The mutation-positive ones were analyzed by DNA sequencing of PCR products. Samples for sequencing were prepared by PCR using BioMix Red (BioLine, Cat# BIO-25006), *slc8b1 seq forward*: 5’-GAGTTTGCCGTTTAATTTCTGG-3’, *slc8b1 seq reverse*: 5’-AATTCCTCACCAAAAAGTGCTC-3’ and 2 μl of gDNA template per 20 μl of reaction. Fish with the confirmed insertions or deletions leading to premature STOP codon were selected as F0 founders. F0 fish was outcrossed with the AB zebrafish line, and their mutation-carrying offspring were in-crossed to generate double-homozygous mutants. Fish were screened via HRM analysis, followed by Sanger sequencing.

### Transient expression of 4mtD3cpv Ca^2+^ probe

To assess basal mitochondrial Ca^2+^ levels in the brain in skeletal muscle of zebrafish embryos in vivo, the genetically encoded FRET-based Ca²⁺ probe 4mtD3cpv was used. The probe was expressed by microinjection of plasmid DNA encoding 4mtD3cpv under the control of the human cytomegalovirus (CMV) promoter^42^ (a kind gift from the laboratory of Prof. Wolfgang F. Graier). Approximately 1 nl of plasmid DNA solution (400 ng/µl) was injected into the cell of one-cell stage embryos to obtain transient and mosaic expression of the probe. Embryos were screened for expression at 1 dpf using fluorescence microscopy. Only those showing appropriate mitochondrial localization of the probe were selected for imaging. Microinjections were performed as described above.

### Quantitative real-time PCR

Zebrafish larvae were euthanized with 0.3 mg/ml Tricaine. Total RNA was extracted from pools of 15–20 larvae or dissected tissues dissected from two adult zebrafish using TRI Reagent (Invitrogen, Cat# AM9738) according to a previously published protocol^81^. RNA concentration and purity were measured using a NanoDrop spectrophotometer. Only samples with absorbance >1.8 at A260/280 nm were used for further processing. Reverse transcription was performed with 500 ng of total RNA using iScript™ cDNA Synthesis Kit (BioRad, Cat# 1708891).

Quantitative PCR was conducted using SsoAdvanced Universal SYBR Green Supermix (BioRad, Cat# 1725274) on a CFX Connect RT-PCR Detection System (Bio-Rad). Primer sequences are listed in Supplementary Table 1. Relative gene expression levels were calculated using the 2^ΔΔCq^ method, with *rpl13a*, *ef1a*, or *18S* used as reference genes depending on tissue type. Each reaction was performed in technical duplicate using 25 ng of cDNA and 0.25 μM of each primer. Changes in expression are expressed as fold changes using expression in WT as a reference value. The calculations were performed in Microsoft Excel.

### Quantification of mitochondrial (mtDNA) to nuclear DNA (nDNA) ratio

DNA was isolated from zebrafish following a previously published protocol^82^. Adult zebrafish were euthanized with 0.3 mg/ml Tricaine and sacrificed to obtain a single DNA sample. The samples were incubated in a lysis buffer (75 mM NaCl, 50 mM EDTA, 20 mM HEPES pH 7.5, 0.4% SDS, 1 mg/ml proteinase K) for at least 4 h in 55 °C. For adult zebrafish, brain and skeletal muscle samples were dissected and homogenized with a plastic pestle in lysis buffer before incubation. DNA was precipitated with isopropanol and incubated for at least 6 h in −20 °C. Following centrifugation, the DNA pellet was washed with 70% ethanol, air-dried, and dissolved in Tris-EDTA (TE) buffer. Relative mitochondrial DNA (mtDNA) abundance was quantified by measuring the levels of *mitochondrial NADH dehydrogenase* (*mt-nd1*) and *nuclear elongation factor 1-alpha* (*ef1a*) using qRT-PCR (primers listed in Supplementary Table 1). A total of 25 ng of DNA was used per qRT-PCR reaction. Changes in the mtDNA-to-nDNA ratio were calculated as fold changes using WT as a reference. Calculations were performed in Microsoft Excel.

### Whole-mount in situ hybridization

Zebrafish larvae were euthanized with 0.3 mg/ml Tricaine. Whole-mount in situ hybridization (WISH) was performed on embryos fixed overnight in 4% paraformaldehyde (PFA) at room temperature. Fixed embryos were dehydrated in a graded methanol series and stored at −20 °C in 100% methanol. Whole-mount in situ hybridization (WISH) was performed as previously described (Soman et al. 2019) with several modifications. Prior to hybridization, embryos were rehydrated through a methanol/PBST (PBS + 0.1% Tween-20) series and treated with 0.1% sodium citrate and 0.1% Triton X-100 for 10 min on ice for permeabilization. Pre-hybridization was performed in Hybridization Solution (HS; 50% deionized formamide, 5X sodium citrate buffer (SSC), 500 μg/ml tRNA, 50 μg/ml heparin, 0.1% Tween-20, 10 mM citric acid, 5% sodium dextran sulfate) at 65 °C for 3 h. DIG-labeled antisense RNA probes were synthesized by in vitro transcription using MegaScript T7 Transcription Kit (Invitrogen, Cat# A57622), digoxigenin (DIG)-labeled UTP (Roche, Cat# 11277073910) and template DNA generated by PCR amplification of target gene sequences. Hybridization was carried out at 67 °C overnight in the hybridization buffer (0.5–3 μg probe/ml buffer). After stringent washes with Hybridization Wash Solution (HWS; 50% deionized formamide, 5X SSC, 0.1% Tween-20, 10 mM citric acid) and subsequently with 75% HWS, 50% HWS, 25% HWS in 2X SSCT (SSC with 0.1% Tween-20) and 0.2X SSCT at 65 °C, embryos were blocked with 10% Blocking Reagent (Roche, Cat# 11096176001) in Maleic Acid buffer (100 mM maleic acid, 100 mM NaCl, 50 mM MgCl_2_, 0.1% Tween-20) and incubated with an alkaline phosphatase-conjugated anti-DIG antibody (1:5000, Roche, Cat# 11093274910) overnight at 4°C. Staining was developed using nitro-blue tetrazolium chloride (0.45 mg/ml, Roche, Cat# 11383213001) and 5-bromo-4-chloro-3-indolyl-phosphate (0.175 mg/ml, Roche, Cat# 11383221001) in Detection buffer (50 mM Tris-HCl pH 9.5, 50 mM NaCl, 25 mM MgCl_2_, 0.05% Tween 20, 2% polyvinyl alcohol) for 3 h and monitored under a stereomicroscope. Background signal was cleaned by 10 min wash with 100% methanol and samples were re-fixed with 4% PFA for 20 min at room temperature and stored in the glycerol until imaging. Images were captured with a Nikon SMZ25 stereomicroscope.

### Histology

Adult zebrafish were euthanized with 0.3 mg/ml Tricaine. For histological analyses, eight-month-old zebrafish were fixed in Bouin’s solution, dehydrated in ethanol, xylene and embedded in paraffin using standard protocols. Embedded samples were sectioned at 6 µm thickness using a Leica microtome (RM2265, Leica). Sections were stained with Hematoxylin and Eosin (H&E) for general tissue morphology, Alcian Blue–Periodic Acid Schiff (AB-PAS) for mucopolysaccharides and glycogen, or Masson’s Trichrome for collagen visualization. Slides were imaged using a Nikon Eclipse 90i microscope with a Nikon DS5-U1 camera (Nikon Corporation, Japan) and the computer image analysis system NIS-Elements AR (Nikon Corporation, Japan). The quantification of Alcian Blue and Masson’s trichrome staining intensity and area was performed using the Colour Deconvolution plug-in in ImageJ.

### Isolation zebrafish larvae mitochondria

Mitochondria were isolated from zebrafish larvae at 5 dpf as previously described by Prudent et al.^83^, with slight modifications. Approximately 300-450 larvae were pooled per biological replicate. Larvae were euthanized with 0.2 mg/ml Tricaine and homogenized in 4 ml of cold isolation buffer (210 mM mannitol, 70 mM sucrose, 1 mM EDTA, 10 mM HEPES, pH 7.5, supplemented with 2 mM PMSF and 2 mg/ml BSA) using a glass-Teflon homogenizer. Homogenates were centrifuged twice at 1500 × g for 10 min at 4 °C to remove debris and nuclei. The supernatant was transferred to a new tube and centrifuged at 14,000 × g for 15 min at 4 °C. The mitochondrial pellet was washed twice in an ice-cold KCl medium (125 mM KCl, 2 mM K_2_HPO_4_, 1 mM MgCl_2_, 5 mM glutamate, 5 mM malic acid, 20 mM HEPES, pH 7) and resuspended in the same buffer. Protein concentration was measured using the Bradford assay (BioRad, Cat# 5000006). Mitochondrial suspensions were kept on ice and used within 1 h after preparation.

### Ca^2+^ measurements in isolated mitochondria

Mitochondrial Ca^2+^ fluxes were measured in freshly isolated mitochondria from 5 dpf zebrafish larvae. 25 µg of mitochondria were gently suspended in KCl medium with glutamate (5 mM), malic acid (5 mM) freshly added, and transferred into a 96-well half-area black polystyrene microplate (Corning®, Cat# CLS3694). Thapsigargin (0.2 µM, Invitrogen™, Cat# T7459) was added in order to inhibit SERCA pump from potential contaminants from the endoplasmic reticulum in the samples. The bath [Ca^2+^] was detected using the Ca^2+^-sensitive dye Oregon Green™ 488 BAPTA-5N hexapotassium salt (2 µM, Invitrogen™, Cat# O6812) as described in Soman et al.^11^. The signal was measured using a plate reader (Tecan Infinite M1000 Pro) at excitation 494 nm/emission 521 nm. Measurements were performed at room temperature.

For the analysis, the first readout of baseline recording was defined as F_0_ and used for normalization. All traces are shown as ΔF/F_0_, where ΔF = F − F_0_. The experimental workflow required temporary suspension of fluorescence acquisition (~40 s) during addition of Ca^2+^, Ru360 and Na^+^ solutions. This caused early uptake/efflux phases to fall outside the recorded interval. Kinetic parameters were therefore calculated only from the measurable portion of the traces, enabling reliable genotype comparisons while not representing absolute transport rates.

Mitochondrial Ca^2+^ level: Mitochondrial Ca^2+^ levels were estimated by measuring the amount of Ca^2+^ released from mitochondria following complete depolarization with CCCP. A 10 µM CCCP (Sigma-Aldrich, Cat# C2759) solution diluted in KCl medium was added using a multichannel pipette after 30 s of baseline recording. Simultaneously, KCl medium without CCCP was added to negative control samples. Fluorescence signals were recorded every 5 s for 5 min. Mitochondrial Ca^2+^ levels were quantified as the maximum ΔF/F_0_ value observed after CCCP addition.

Mitochondrial Ca^2+^ uptake assay: 20 µM CaCl_2_ diluted in KCl medium was the signal was measured every 3 s for 10 min. Ca^2+^ uptake was calculated as the difference between the maximal ΔF/F_0_ (first read-out after CaCl_2_ addition) and ΔF/F_0_ value measured 2 min after CaCl_2_ addition.

Na^+^-dependent mitochondrial Ca^2+^ efflux: Mitochondria were first loaded with 20 µM CaCl_2_ diluted in KCl medium, as described for the mitochondrial uptake assay. Next, mitochondrial Ca^2+^ uptake via MCU was inhibited by adding 10 µM Ru360 (Sigma-Aldrich, Cat# 557440). To estimate the amount of Na^+^-independent Ca^2+^ efflux following Ru360 addition, the last ΔF/F_0_ value recorded after CaCl_2_ addition was subtracted from the maximum ΔF/F_0_ value obtained after Ru360 addition. Subsequently, Na^+^-dependent mitochondrial Ca^2+^ efflux was stimulated by adding 10 mM NaCl (KCl medium in which 120 mM K^+^ was substituted with Na^+^). To estimate the amount of Ca^2+^ efflux following Na^+^ addition, the last ΔF/F_0_ value recorded after Ru360 addition was subtracted from the maximum ΔF/F_0_ value after Na⁺ addition. Fluorescence signals were recorded every 5 seconds for 5 minutes following each treatment.

### In-gel complex I activity assay

Zebrafish larvae were euthanized with 0.3 mg/ml Tricaine and snap-frozen in liquid nitrogen and stored at -80 °C until mitochondria isolation. Mitochondria (200 µg total protein) were pelleted by centrifugation and resuspended in 20 µl of buffer A (50 mM NaCl, 50 mM imidazole, pH 7.0, 1 mM EDTA, 2 mM 6-aminocaproic acid). Samples were solubilized with 6 µL of 20% (w/v) digitonin (detergent-to-protein ratio of 6 g/g) and centrifuged at 20,000 × *g* for 20 min at 4 °C. The supernatant protein concentration was determined, and 100 µg of solubilized protein was loaded onto 3–18% gradient gels for Blue Native Electrophoresis (BNE) as described previously^84^.

In-gel Complex I activity (NADH:NTB reductase) was visualized by incubating gels in assay buffer containing 2.5 mg nitroblue tetrazolium (NTB) and 10 µL NADH (10 mg/mL) in 1 mL of 5 mM Tris-HCl (pH 7.4)^85^. The reaction was stopped with 50% (v/v) methanol and 10% (v/v) acetic acid and rinsed in distilled water^86^.

### NADP to NADPH ratio

The NADP/NADPH ratio was determined using the NADP/NADPH Assay Kit (Abcam, Cat# ab65349) following the manufacturer’s instructions. Zebrafish larvae were euthanized with 0.3 mg/ml Tricaine. For each biological replicate, 100 larvae were pooled and homogenized in 500 µl Extraction Buffer using a pestle. Homogenates were centrifuged at 18,000 g for 15 min at 4 °C, and the supernatants were passed through 10 kDa Spin Columns (Abcam, Cat. #ab93349) to remove NADPH-consuming enzymes by centrifugation at 10,000 g for 15 min at 4 °C.

Each sample was split into two aliquots: one left untreated to quantify total NADP + NADPH, and another heated at 60 °C for 30 min to decompose NADP for NADPH-only measurement. Samples were diluted 1:5 in Extraction Buffer, and 100 µl of Reaction Mix (98 µl NADP Cycling Buffer + 2 µl NADP Cycling Enzyme Mix) was added, followed by 5 min incubation at room temperature. Subsequently, 10 µl of Developer Solution was added, and samples were incubated for 1 h at room temperature. Absorbance was measured at 450 nm using a plate reader (Tecan Infinite M1000 Pro). NADPH concentrations were calculated from a standard curve and expressed relative to wild-type levels.

### Spectrophotometric assays of electron transport chain enzyme activities

Zebrafish larvae were euthanized with 0.3 mg/ml Tricaine and snap-frozen in liquid nitrogen and stored at -80 °C until mitochondria isolation. Mitochondrial enzyme activities were determined spectrophotometrically according to^87^ with minor modifications. Isolated mitochondria were resuspended in 10 mM Tris/HCl (pH 7.4) and subjected to two freeze–thaw cycles to permeabilize membranes. All assays were performed in a total reaction volume of 200 µL per well using a microplate spectrophotometer.

Complex I + III (NADH–cytochrome c oxidoreductase): Reaction mixtures contained 5 µM oxidized cytochrome *c*, 1 mg/mL BSA, and 25 µg/µL mitochondrial membranes in 100 µl. The reaction was initiated by adding 100 µl of 150 µM NADH in 10 mM Tris/HCl buffer (pH 7.4) and absorbance was recorded at 340 nm for 5 min. The reaction was terminated by adding 10 µM rotenone.

Complex II (Succinate dehydrogenase): Reaction mixtures contained 5–9 µg/µL mitochondrial membranes, 250 µM KCN, and 10 mM succinate in 100 µl. The reaction was initiated by adding 100 µl of 80 µM 2,6-dichlorophenolindophenol (DCPIP), 1 mg/ml BSA, and 70 µM decylubiquinone (DUQ) in 10 mM Tris/HCl (pH 7.4) and reduction of DCPIP was monitored at 600 nm for 5 min. The reaction was terminated by adding 10 mM malonate.

Complex III (Cytochrome c reductase): Reaction mixtures contained 2–5 µg/µl mitochondrial membranes, 75 µM oxidized cytochrome *c*, 250 µM KCN, and 100 µM EDTA in 100 µl. The reaction was initiated by adding 100 µl of 1 mg/ml BSA and 100 µM decylubiquinol (DUQH₂) in 25 mM potassium phosphate buffer (pH 7.4) and absorbance at 550 nm was recorded for 5 min. The reaction was stopped by adding 10 µg/ml antimycin A.

Complex IV (Cytochrome c oxidase): Reaction mixtures contained 2–5 µg/µl mitochondrial membranes and 75 µM reduced cytochrome c in 25 mM potassium phosphate buffer (pH 7.4). Absorbance at 550 nm was measured for 5 min after reaction initiation. The reaction was terminated with 150 µM KCN.

Citrate synthase (CS): Citrate synthase activity was used as a marker for mitochondrial content. The reaction mixture contained 1–10 µg/µL mitochondrial protein, 0.1% Triton X-100, 100 µM 5,5′-dithiobis-(2-nitrobenzoic acid) (DTNB), and 300 µM acetyl-CoA in 200 mM Tris/HCl buffer (pH 8.0) in 190 µl. The reaction was initiated by adding 500 µM oxaloacetate, and the formation of the thionitrobenzoate anion was monitored at 412 nm for 5 min.

### ATP content

ATP content in zebrafish larvae was determined using the ATP Bioluminescence Assay Kit HS II (Sigma-Aldrich, Cat# 11699709001) following the manufacturer’s instructions. Briefly, ten 5-day post-fertilization (dpf) larvae per biological replicate were collected on ice and lysed in 100 µl Lysis Buffer. Samples were heated at 95 °C for 2 min and centrifuged at maximum speed for 1 min at 4 °C. The clear supernatant was diluted 1:5 in Dilution Buffer, and 2.5 µl of the diluted sample was mixed with 187.5 µl of Dilution Buffer and 10 µl of luciferase reagent. Luminescence was recorded immediately using a microplate reader (Tecan Spark Multimode Microplate Reader). ATP concentrations were calculated from a standard curve and normalized to protein content determined by the BCA assay.

### Coenzyme Q10 (CoQ10) determination

CoQ extraction: CoQ extraction from zebrafish larvae was performed as previously described^88^ with minor modifications. Zebrafish larvae were euthanized with 0.3 mg/ml Tricaine. For each biological replicate, 50 larvae were pooled, washed with ice-cold PBS, and transferred into pre-chilled lysis tubes (Lysing Matrix S, MP Biomedicals, Cat# 116925050). Samples were homogenized in a mixture of 250 µl ice-cold methanol containing 0.1% hydrochloric acid and 250 µl ice-cold hexane using a Bullet Blender (MP Biomedicals). The homogenate was transferred to fresh microcentrifuge tubes and centrifuged at 17,000 g for 5 min at 4 °C to separate the CoQ-containing hexane phase. The upper layer was collected and evaporated to dryness under a stream of nitrogen in 24-well plates. Dried extracts were resuspended in ice-cold methanol, transferred to HPLC vials, overlaid with nitrogen, and stored at –80 °C until analysis.

CoQ10 measurements: Quantification of oxidized (CoQ10) and reduced (CoQ10H_2_) forms was performed by high-performance liquid chromatography with electrochemical detection (HPLC-ECD), as described previously^89^ with minor modifications. Separation was achieved on a Microsorb-MV 100-5 C18 reversed-phase column (150 × 4.6 mm, 5 µm bead size; Agilent, Cat# AG-R0086200D5) at room temperature with a flow rate of 1.1 ml/min. The mobile phase consisted of 4.2 g sodium acetate anhydrous, 15 ml acetic acid, 15 ml isopropanol, 140 ml hexane, and 830 ml methanol in a total volume of 1 l. Electrochemical detection was performed using sequential potentials of –850 mV (applied three times to induce CoQ10H_2_ oxidation), +50 mV, +350 mV, +450 mV, +500 mV, and +550 mV. CoQ10 and CoQ10H_2_ yielded maximal responses at +350 mV, with typical retention times of 12.3 min and 7.1 min, respectively. Commercial CoQ10 (Cayman Chemical, Cat# CAY11506-200) and chemically reduced CoQH_2_ (prepared in-house by sodium borohydride reduction^88^) were used for peak identification and quantification.

### Catalase activity

Catalase activity was measured using the Catalase Activity Assay Kit (Abcam, Cat# ab83464) according to the manufacturer’s protocol, with minor modifications. Zebrafish larvae were euthanized with 0.3 mg/ml Tricaine. For each biological replicate, 100 zebrafish larvae were collected, washed in cold PBS, and homogenized on ice in 200 µl of ice-cold Catalase Assay Buffer using a pestle. The homogenates were centrifuged at 10,000 g for 15 min at 4 °C, and the supernatants were transferred to fresh tubes.

Assays were performed in 96-well half-area black polystyrene microplate (Corning®, Cat# CLS3694) using 50 µl total volume per well. Samples (39 µl) were incubated with 6 µl of 125 µM H₂O₂ in Catalase Assay Buffer at 25 °C for 30 min. Reactions were stopped by adding 5 µl of Stop Solution, followed by addition of 25 µl Developer Mix containing Catalase Assay Buffer, OxiRed Probe, and HRP solution. After 10 min incubation at 25 °C in the dark, fluorescence was measured at Ex/Em = 535/587 nm using a plate reader (Tecan Infinite M1000 Pro). Hydrogen peroxide standards were prepared freshly to generate a standard curve. Catalase activity was calculated based on the rate of H_2_O_2_ decomposition and expressed relative to wild-type levels.

### TMRE microinjections

The stock solution of tetramethylrhodamine ethyl ester (TMRE; Invitrogen™, Cat# T669) was prepared by dissolving in dimethyl sulfoxide (DMSO, Sigma-Aldrich, Cat# D8418) to a final concentration of 10 mM. The injection solution was prepared at the day of the experiment by diluting the 1:5 stock solution in water (final concentration: 2 mM) and 1 nl was injected into the yolk of embryos at the 1-2 cell stage. Microinjections were performed in the same way as for the generation of *nclx*^−/−^ mutants. Embryos were then grown according to standard methods described in the “Animal maintenance” section.

### In vivo imaging with LSFM

Stained or transgenic larvae were anesthetized with 0.2 mg/ml Tricaine, mounted in 2% low-melting-point agarose (Sigma-Aldrich, Cat# A9414) in a glass capillary and imaged under a Zeiss Lightsheet Z.1 microscope using a 40X objective (Zeiss) with excitation at 488 nm (AO, *Tg*(*CEPIA2mt*) and CellROX), 561 nm (TMRE) or 445 nm (4mtD3cpv). Fish were imaged from the dorsal side of the head and were pushed from the capillary to be submerged in the medium. Measurements were performed at room temperature.

Images were first processed in ZEN software (Zeiss) in order to obtain merged maximum projection images, exported to TIF files and analyzed further with ImageJ.

Cell death estimation by Acridine Orange in vivo staining: Acridine Orange (AO; Sigma-Aldrich, Cat# A8097) was added to the plates containing live 4 dpf zebrafish larvae (final concentration: 10 µg/ml in E3). After 1 hour of incubation in the dark, the medium was exchanged with fresh E3 six times during a period of 30 min. Z-stacks encompassing 45 µm of the optic tectum with 5 µm interval were acquired with a Zeiss Lightsheet Z.1 microscope. The counting of the AO-stained cells in the optic tectum of each larvae, which were considered as dying^90^, was performed manually on the maximum projection images with the CellCounter plug-in in ImageJ. The number of cells was normalized to the analyzed area of the optic tectum.

Mitochondrial Ca^2+^ flux alive zebrafish with measured with CEPIA2mt: In order to estimate mitochondrial Ca^2+^ content 5 dpf larvae that expressed CEPIA2mt, Ca^2+^ probe targeted to the mitochondria under neural promoter *HuC*, were treated with 10 μM CCCP (Sigma-Aldrich, Cat# C2759) to induce depolarization of mitochondria and release of mitochondrial Ca^2+^. After mounting the zebrafish larvae in the microscopic chamber filled with E3 the medium in the chamber was exchanged to E3 with 10 μM CCCP. To gain complete depolarization, larvae were treated for 10 min. Z-stacks encompassing 10 µm of optic tectum with 1 µm interval were acquired with Zeiss Lightsheet Z.1 microscope every 5 s during 10 min of treatment.

During image analysis The StackReg plug-in was applied for movement correction. Mitochondria were detected by thresholding and mean fluorescence for mitochondria in the selected square ROI in the optic tectum was calculated. Background fluorescence was calculated as the mean fluorescence of a square that was localized besides the fish, and subtracted from the mean fluorescence of ROI value: F = F_ROI_ - F_background_. F was normalized by the value obtained after full depolarization (F/F_CCCP_) and the ratio between basal fluorescence (F_0_) and F_CCCP_ was calculated to compare mitochondrial Ca^2+^ content between variants similarly to the method described in ref. ^68^.

Reactive oxygen species (ROS) quantification: ROS levels were assessed in vivo using CellROX™ Green Reagent (Invitrogen, Cat# C10444). 5 dpf zebrafish larvae were incubated in E3 medium containing 5 µM CellROX Green for 1 h in the dark at 28 °C. After staining, larvae were washed by exchanging the medium with fresh E3 six times over 30 minutes to remove unbound dye. Imaging was performed using a Zeiss Lightsheet Z.1 microscope, acquiring Z-stacks spanning 50 µm of the optic tectum with 10 µm intervals. Mean fluorescence intensity was quantified for each larva and used as a measure of relative ROS levels.

Basal mitochondrial Ca^2+^ measured in vivo with 4mtD3cpv: the use of the FRET-based Cameleon 4mtD3cpv allowed for ratiometric imaging and enabled estimation of basal Ca^2+^ levels, independent of probe expression level^43,44^. Brains and somites of 2 dpf dechorionated larvae injected with the pcDNA3-4mtD3cpv vector were imaged using a Zeiss Lightsheet Z.1 microscope. The intensities of cyan fluorescent protein (CFP, emission: 460–500 nm) and circularly permuted Venus (cpV, emission: 522-565 nm) were recorded every 12 s for 1 min in a z-stack of 10 µm with 1 µm intervals.

During image analysis, the StackReg plug-in was used for motion correction. Cells in the skeletal muscle cells of the optic tectum that showed 4mtD3cpv expression were manually selected as ROIs. Mitochondria were detected by thresholding, and mean CFP and cpV fluorescence were quantified in each ROI. The mean ratio of cpV to CFP was calculated for the brain and muscles of each larva.

Mitochondrial membrane potential (MMP) in vivo measurements: Two days after microinjections with TMRE dechorionated larvae were imaged using lightsheet fluorescence microscopy (LSFM) as described above. 2 dpf larvae were treated with 10 μM CCCP (Sigma-Aldrich, Cat# C2759) to induce depolarization of mitochondria. To gain complete depolarization, larvae were treated for 5 min. Z-stacks encompassing 10 µm of the optic tectum with 1 µm intervals were acquired with a Zeiss Lightsheet Z.1 microscope every 5 s during 5 min of treatment. F was normalized to the value obtained after full depolarization (F/F_CCCP_) and the ratio between basal fluorescence (F_0_) and F_CCCP_ was calculated to compare mitochondrial membrane potential between variants. Image analysis was performed as for mitochondrial Ca^2+^ measurements described above.

### Coiling activity of zebrafish embryos

Randomly selected embryos were transferred to a 12-well plate (4 embryos/well) in 2 ml of E3 and acclimated for 2 h in 28 °C. Coiling activity was recorded at 30 hpf using the Nikon SMZ25 stereomicroscope for 3 min with a 10 frames /s acquisition rate. The number of coiling events for each embryo was counted manually.

### Transmission electron microscopy

Zebrafish larvae at 5 dpf were euthanized with 0.3 mg/ml Tricaine. The animals were fixed in 2.5% glutaraldehyde for 24 h at 4 °C, washed in PBS, postfixed in 1% osmium tetroxide for 1 h, washed with water, and stained with 1% aqueous uranyl acetate overnight at 4 °C. The larvae were dehydrated and infiltrated with epoxy resin (Sigma Aldrich, Cat# 45-359-1EA-F). Samples were then polymerized at 60 °C for 48 h. Polymerized blocks were trimmed with a tissue processor (Leica EM TP) and cut with an ultramicrotome (EM UC7, Leica) to obtain ultrathin sections (70 nm thick), which were collected on nickel grids (200 mesh, Agar Scientific, Cat# G2200N). The grids were examined using a Tecnai T12 BioTwin transmission electron microscope (FEI) equipped with a 16-megapixel TemCam-F416 camera (TVIPS GmbH) at the Microscopy and Cytometry Facility at IIMCB in Warsaw.

### Statistics and reproducibility

All statistical analyses were conducted using R version 4.1.2. Box and whisker plots were generated with R, while other graphs were generated with Microsoft Excel or GraphPad Prism version 8. All datasets were tested for outliers using a Grubbs’ test and for normal distribution using the Shapiro-Wilk test. The applied statistical tests, sample sizes and replicates number for each experiment are indicated in the figure legends.

### Reporting summary

Further information on research design is available in the Nature Portfolio Reporting Summary linked to this article.

## Supplementary information

Supplementary Information

Description of Additional Supplementary Files

Supplementary Data 1

Reporting Summary

## Data Availability

This study includes no data deposited in external repositories. No unbiased larger data sets were generated in this study. All data are presented in the results or as supplemental data. The source data behind the graphs presented in the study can be found in the Supplementary Data 1. All unique zebrafish lines generated in the study are available with a completed materials transfer agreement. Any additional information required to reanalyze the data reported in this paper is available upon request.
