# Supplementary material for: Tmbim5 and Slc8b1 cooperate in tissue-specific mitochondrial calcium regulation in zebrafish
Source: Commun Biol. 2026 Jan 8;9:218. doi: 10.1038/s42003-025-09494-7 (PMC12894878; doi:10.1038/s42003-025-09494-7)
Supplement: Supplementary file 1 — Supplementary Information [file 42003_2025_9494_MOESM1_ESM.pdf]

# Supplementary information

## Tables

**Supplementary Table 1. Primers that were used for riboprobe synthesis and qRT-PCR**
All primers' sequences are listed as from 5' to 3'.

| Gene name | Abbreviation | Primer sequence | |
|---|---|---|---|
| | | Forward | Reverse |
| **qRT-PCR** | | | |
| *transmembrane BAX inhibitor motif-containing protein 5* *(growth hormone inducible transmembrane protein)* | *tmbim5 (ghitm)* | GTCTCGGCATGTCCAGTGAG | CATGATCCCCATGAGCACCG |
| *solute carrier family 8 member B1* | *slc8b1* | CGGAGTTCACACGGACCTAC | GATGGCTAGGGGCAGAAGAC |
| *eukaryotic translation elongation factor 1 α1, like 1* | *ef1a* | AAAATCGGTGGTGCTGGCAA | GGAACGGTGTGATTGAGGGA |
| *ribosomal protein L13a* | *rpl13a* | TCTGGAGGACTGTAAGAGGTATGC | AGACGCACAATCTTGAGAGCAG |
| *18S ribosomal RNA* | *18S* | TCGCTAGTTGGCATCGTTTATG | CGGAGGTTCGAAGACGATCA |
| *mitochondrial calcium uniporter* | *mcu* | GTATCCCGCATTCGGTGTCT | CTGTTCTCAGACCGTGTGCT |
| *mitochondrial calcium uptake 1* | *micu1* | GACGGAGAGGTTGACCTTGA | GCGATCTCGGTGTCTCATCC |
| *mitochondrial calcium uptake 2* | *micu2* | ACCAAAGCAGACTTGGACGAA | GATCAACCCCTTGTCACCGA |
| *mitochondrial calcium uptake 3* | *micu3* | TGCCGTTCCATTGGACAAGA | TGGTGTTAACCAGGTGTCGG |
| *EF-hand domain family member d1* | *efhd1* | ATCGACGTGTCCACTGAAGG | TCAGCCTCGAACTTACTGCG |
| *leucine zipper-EF-hand containing transmembrane protein 1* | *letm1* | GGTGGATGCAGAACACGCT | TCGCTCAGCATGTCAATCTCC |
| *leucine zipper-EF-hand containing transmembrane protein 2* | *letm2* | CCGATCTGACACCATACCCG | TCAGGCGTCCTGTCGAAAAA |
| *LETM1 domain-containing protein 1* | *letmd1* | CTAGATTGGCTTCCTCAAAACCCAG | CAGGAGTCTGAAGCCTCTCAT |
| *solute carrier family 8 member B1* | *slc8b1* | CGGAGTTCACACGGACCTAC | GATGGCTAGGGGCAGAAGAC |
| *transmembrane Protein 65* | *tmem65* | CAACGCCATTCCCTTCATCG | TTCAATCTGAGTGCCAGCGG |
| *solute carrier family 25 member 28 (mitoferrin-2)* | *slc25a28* | ACCGGACTATGAGGGTTTGC | AGGCTCTGCATACGTGTCTTT |

| | | | |
|---|---|---|---|
| *AFG3-like AAA ATPase 2* | *afg3l2* | CTCTAATGAGGCCTGGACGC | CGATGTCCGCACCTGAAAAA |
| *mitochondrial NADH dehydrogenase 1* | *mt-nd1* | AGCCATCTCAAGCCTAGCAG | ATTGTTTGCGCTACAGCTCG |
| **riboprobes synthesis for WISH** | | | |
| *transmembrane BAX inhibitor motif-containing protein 5* *(growth hormone inducible transmembrane protein* | *tmbim5* | CGCTGCTCAGAGGTGAAGGT | TAATACGACTCACTATAGGGATGT GTGCGGCATCTCTGCG |
| *solute carrier family 8 member B1* | *slc8b1* | GTGTAGACGTGCGTTCTTGC | TAATACGACTCACTATAGGGACGA GACGCCACAGTAAA |

# Normal behavior of *tmbim5*$^{-/-}$ zebrafish

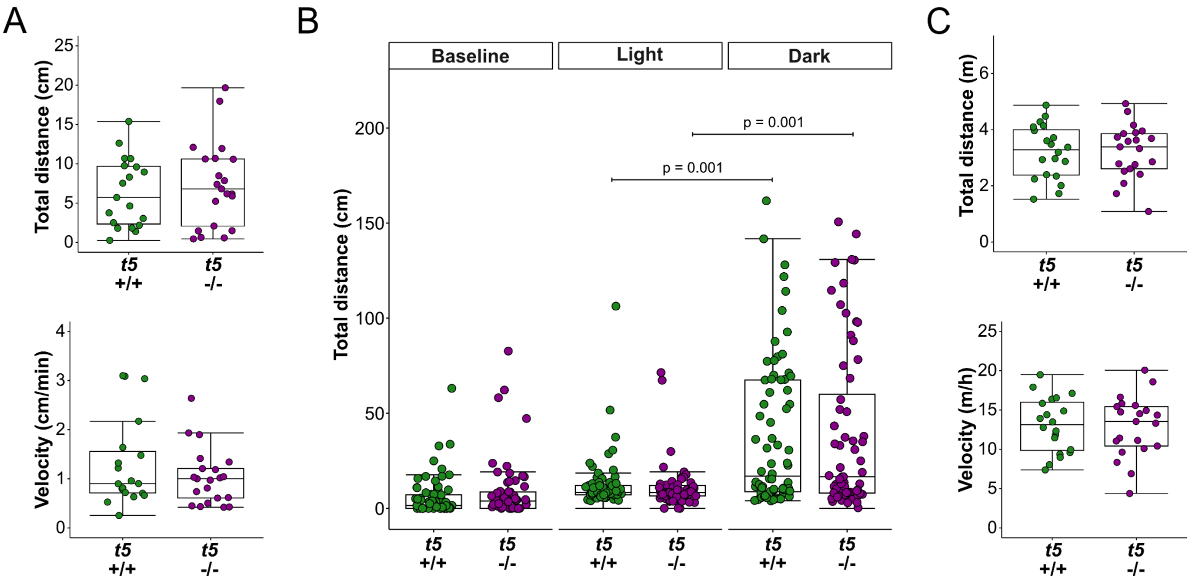


**Supplementary Figure 1. Normal behavior of *tmbim5*$^{-/-}$ zebrafish.**

A) Locomotor activity of 5 dpf larvae in an open-field test is not affected by *tmbim5* knockout (KO). Total distance traveled and mean velocity are plotted as box-and-whisker plots (box: 25th–75th percentile; whiskers: min to max), with each dot representing an individual larva (*n* = 19–21). Number of experiments = 3. Statistical analysis: *t*-test (total distance) or Mann–Whitney test (velocity).

B) Normal visual-motor response of 5 dpf *tmbim5*$^{-/-}$ larvae. Total distance covered during each phase of the experiment is plotted as box-and-whisker plots, with each dot representing an individual larva (*n* = 64–72). Number of experiments = 3. Statistical analysis: Kruskal-Wallis test followed by Dunn's test.

C) Locomotor activity of adult (8-month-old) fish in a novel tank test is not affected by *tmbim5* KO. Total distance traveled and mean velocity are plotted as box-and-whisker plots, with each dot representing an individual fish (*n* = 20–21). Number of experiments = 3. Statistical analysis: Mann–Whitney test.

## Histology of brain, muscle and liver of adult *tmbim5*$^{-/-}$ fish

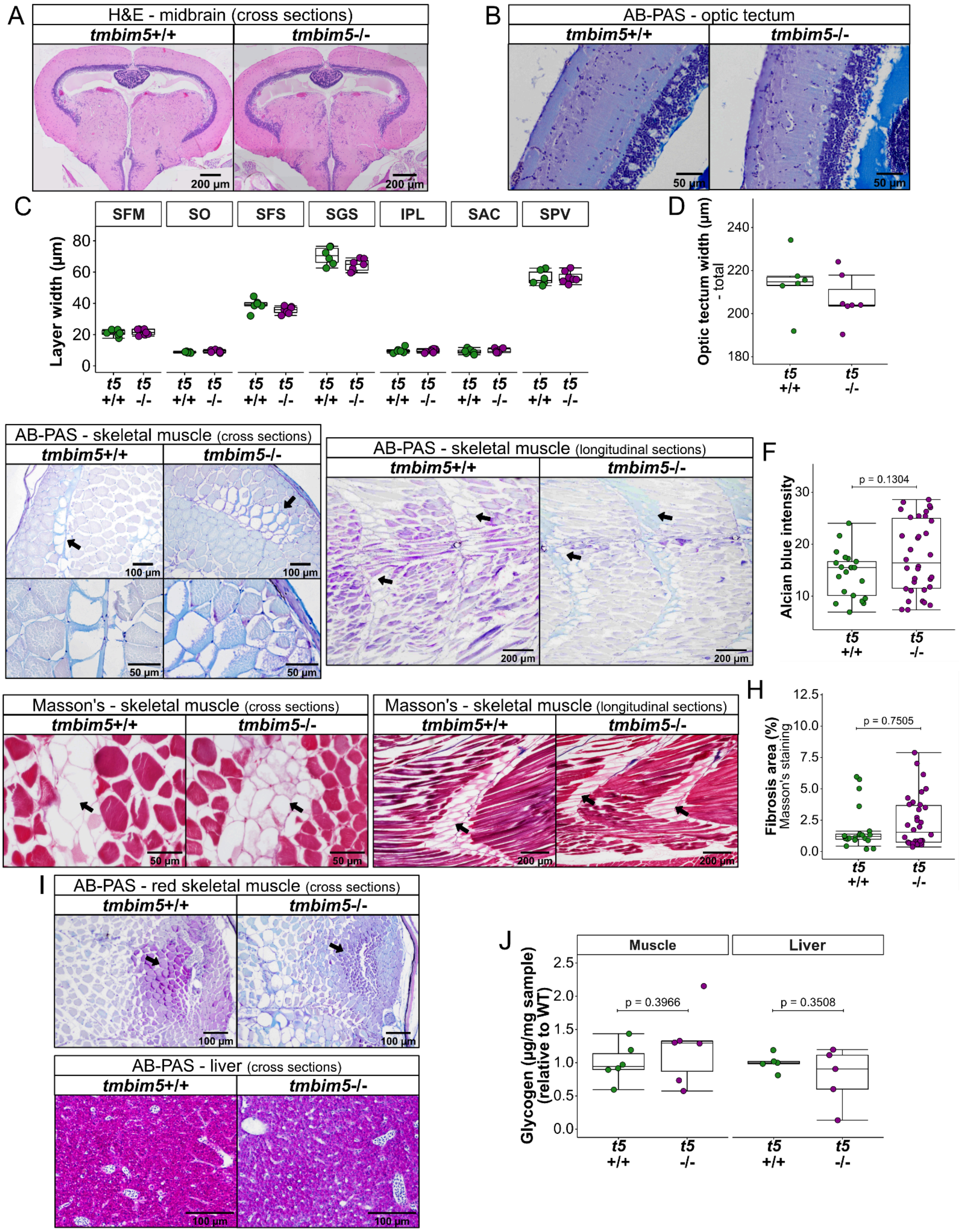


**Supplementary Figure 2. Histology of brain, muscle and liver of adult *tmbim5*$^{-/-}$ fish**

A-D) Normal histology of the *tmbim5*$^{-/-}$ fish brain. A) Representative images of cross-sections of the WT and *tmbim5*$^{-/-}$ midbrain stained with H&E. B) Representative images of cross-sections of the WT and *tmbim5*$^{-/-}$ optic tectum stained with AB-PAS. C) Quantification of the width of optic tectum layers. D) Comparison of total optic tectum width between WT and *tmbim5*$^{-/-}$ fish, showing no significant differences. Data are presented as box-and-whisker plots (box: 25th–75th percentile; whiskers: min to max), with each dot representing an individual fish (5–7 slices analyzed per fish, 5 measurements per slice, $n$ = 6–7). Statistical analysis: $t$-test with BH correction for multiple comparisons. SFM – Stratum Fibrosum Superficiale, SO – Stratum Opticum, SFS – Stratum Fibrosum Superficiale, SGS – Stratum Fibrosum Griseum, IPL – Internal Plexiform Layer, SAC – Stratum Album Centrale, SPV – Stratum Periventriculare.

E-F) Enhanced Alcian Blue (AB) staining in the intermuscular spaces of *tmbim5*$^{-/-}$ fish. E) Representative images of cross- and longitudinal sections of WT and *tmbim5*$^{-/-}$ fish stained with AB-PAS (Periodic Acid-Schiff). Lower panels show magnified views of AB-stained intermuscular spaces (indicated by arrows). F) Quantification of AB staining intensity. Data are presented as box-and-whisker plots, showing a trend toward increased staining intensity in *tmbim5*$^{-/-}$ fish. Each dot represents the average for one slice ($n$ = 21–34, number of fish = 7). Statistical analysis: Mann–Whitney test.

G-H) Masson's trichrome staining of skeletal muscle reveals no differences in *tmbim5*$^{-/-}$ fish. G) Representative images of cross- and longitudinal sections of WT and *tmbim5*$^{-/-}$ fish. Intermuscular spaces that showed positive AB staining are marked with arrows. H) Quantification of Masson's trichrome-positive areas, showing no significant changes in *tmbim5*$^{-/-}$ fish. Each dot represents the average for one slice ($n$ = 21–34, number of fish = 7). Statistical analysis: Mann–Whitney test.

I) Reduced PAS staining intensity in the muscle and liver of *tmbim5*$^{-/-}$ adult (8-month-old) fish. Representative images of cross-sections of WT and *tmbim5*$^{-/-}$ fish stained with AB-PAS.

J) Glycogen levels remain unchanged in the muscle and liver of *tmbim5*$^{-/-}$ adult (1.5-year-old) fish. Quantification was performed using a colorimetric glycogen assay. Each dot represents an independent biological replicate ($n$ = 5–6, number of experiments = 2). Statistical analysis: *t*-test.

## Reduced complex I activity in tmbim5$^{-/-}$ larvae

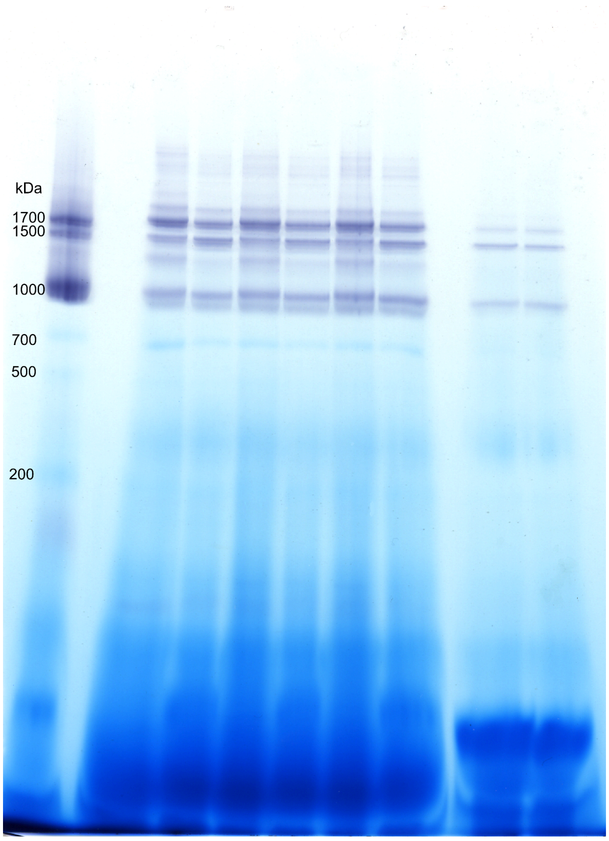


**Supplementary Figure 3. Uncropped and unedited image of blue native gel electrophoresis (BNGE) followed by in-gel complex I activity assay.**

The first lane shows bovine heart mitochondria, which was used as a positive control and the weight marker. The last two lanes show samples with a very low protein concentration, which were excluded from the analysis.

# Generation of Slc8b1-deficient fish

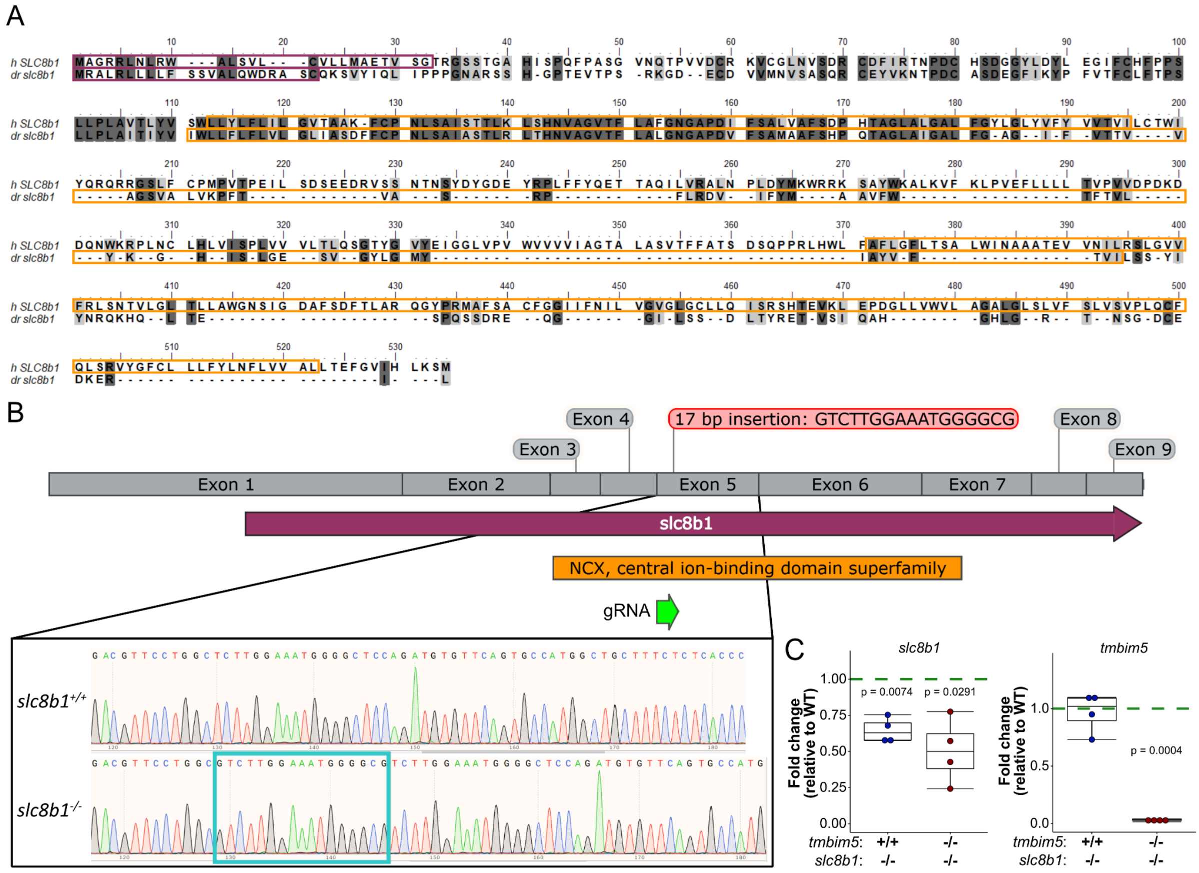


**Supplementary Figure 4. Generation of Slc8b1-deficient fish**

A) Sequence alignment of human and zebrafish NCLX proteins encoded by *slc8b1*, performed using Optimal Global Alignment with the BLOSUM62 similarity matrix. The alignment revealed 24% sequence identity between species. Identical residues are highlighted in dark gray, while residues with similar physico-chemical properties are marked in light gray. The predicted signal peptide is enclosed within a violet frame, and the sodium/calcium ($Na^+/Ca^{2+}$) domain is highlighted by an orange frame.

B) Schematic representation of the mutation site in *slc8b1*, accompanied by Sanger sequencing results. Inserted base pairs are highlighted with a turquoise frame.

C) Expression levels of *slc8b1* and *tmbim5* mRNA in 5 dpf *tmbim5*$^{+/+}$*;slc8b1*$^{-/-}$ and *tmbim5*$^{-/-}$*;slc8b1*$^{-/-}$ larvae, quantified using qPCR and normalized to wild-type. *rpl13a* and *ef1a* were used as reference genes. Results are presented as box-and-whisker plots (box: 25th–75th percentile, whiskers: min–max), with each dot representing an independent biological replicate ($n$ = 4, each RNA sample isolated from 30 larvae). Statistical analysis: one-sample $t$-test with BH correction for multiple comparisons.

## Normal behavior of *slc8b1*$^{-/-}$ and *tmbim5*/*slc8b1* double knockout larvae

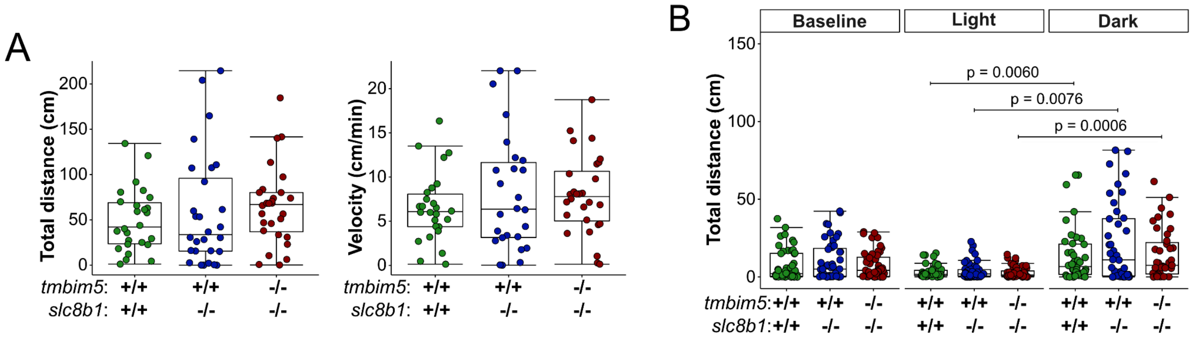


**Supplementary Figure 5. Normal locomotor activity of *slc8b1*$^{-/-}$ and *tmbim5*/*slc8b1* double knockout larvae**

A) Locomotor activity of 5 dpf larvae in an open-field test is not affected by *slc8b1* KO or *tmbim5*$^{-/-}$;*slc8b1*$^{-/-}$ double KO. Total distance traveled and mean velocity are plotted as box-and-whisker plots (box: 25th–75th percentile, whiskers: min–max), with each dot representing an individual larva (*n* = 26–28). Statistical analysis: Kruskal-Wallis test followed by Dunn's post-hoc test. Number of experiments = 2.

B) Normal visual-motor response of *slc8b1*$^{-/-}$ and *tmbim5*$^{-/-}$;*slc8b1*$^{-/-}$ larvae at 5 dpf. Total distance traveled during each phase of the experiment is presented as box-and-whisker plots, with each dot representing an individual larva (*n* = 40–48). Statistical analysis: two-way ANOVA followed by Kruskal-Wallis test and Dunn's post-hoc test. Number of experiments = 2.

## Supplementary Methods

### Glycogen levels quantification

Glycogen levels in the muscles and liver of adult (1.5-year-old) zebrafish were quantified using a colorimetric glycogen assay (Abcam, Cat# ab169558) according to the manufacturer's instructions. Briefly, the assay is based on glycogen hydrolysis into glucose, which is oxidized to form an intermediate that reduces a colorless probe to a colored product with strong absorbance at 450 nm. Fish were euthanized with 0.3 mg/ml Tricaine and dissected to obtain liver and muscle samples. Tissue was weighed before homogenization using a glass-Teflon homogenizer on ice. Glycogen levels were estimated by measuring the optical density (OD) at 450 nm using an absorbance microplate reader (Tecan Sunrise). Samples that were not treated with the glycogen-hydrolyzing enzyme served as background controls. Glycogen content was normalized to tissue weight and expressed as a fold change using WT as a reference.

### Behavioral analysis

Open field test: Randomly selected 4 dpf larvae were acclimated to the behavioral testing room for at least 15 min. Two minutes before recording locomotor activity, the larvae were transferred to a 12-well plate that was then placed in the ZebraBox, a high-throughput monitoring system (ViewPoint). The experiment was performed in a volume of 2 mL of E3 medium, and the light intensity was set to 70%. Locomotor activity was recorded for 10 min.

Visual motor response: The experiment was performed according to the procedure described previously [1]. On the day before the experiment, the larvae were placed in 24-well plates that contained 0.5 mL of E3 medium. Thirty minutes before recording locomotor activity, the plates were placed in the ZebraBox. The experiment consisted of three phases of the following changes in lighting conditions: baseline (0–10 min, 0% light intensity), light (10–20 min, 70% light intensity), and dark (20–30 min, 0% light intensity).

Novel tank test for adult zebrafish: The 8-month-old zebrafish were acclimated to the behavioral testing room for one week before the experiment. Fish were placed individually in a transparent tank (24 cm length x 14 cm height x 6cm width) filled with aquarium water. ZebraCube, an adult fish monitoring system (ViewPoint) was utilized to record animals' behavior for 15 min.

The video files acquired during experiments with larvae and adult zebrafish were further analyzed using EthoVision XT software (Noldus).

## Supplementary References


1. Kedra, M. *et al.* TrkB hyperactivity contributes to brain dysconnectivity, epileptogenesis, and anxiety in zebrafish model of Tuberous Sclerosis Complex. *Proc. Natl. Acad. Sci. U. S. A.* **117**, 2170–2179 (2020).
