# Supplementary material for: Tmbim5 and Slc8b1 cooperate in tissue-specific mitochondrial calcium regulation in zebrafish
Source: Commun Biol. 2026 Jan 8;9:218. doi: 10.1038/s42003-025-09494-7 (PMC12894878; doi:10.1038/s42003-025-09494-7)
Supplement: Supplementary file 4 — Reporting Summary [file 42003_2025_9494_MOESM4_ESM.pdf]

nature portfolio

Corresponding author(s): Axel Methner

Last updated by author(s): Nov 30, 2025

Reporting Summary

Nature Portfolio wishes to improve the reproducibility of the work that we publish. This form provides structure for consistency and transparency in reporting. For further information on Nature Portfolio policies, see our Editorial Policies and the Editorial Policy Checklist.

Statistics

For all statistical analyses, confirm that the following items are present in the figure legend, table legend, main text, or Methods section.

n/a	Confirmed	
☐	☒	The exact sample size (*n*) for each experimental group/condition, given as a discrete number and unit of measurement
☐	☒	A statement on whether measurements were taken from distinct samples or whether the same sample was measured repeatedly
☐	☒	The statistical test(s) used AND whether they are one- or two-sided<br>*Only common tests should be described solely by name; describe more complex techniques in the Methods section.*
☒	☐	A description of all covariates tested
☐	☒	A description of any assumptions or corrections, such as tests of normality and adjustment for multiple comparisons
☐	☒	A full description of the statistical parameters including central tendency (e.g. means) or other basic estimates (e.g. regression coefficient) AND variation (e.g. standard deviation) or associated estimates of uncertainty (e.g. confidence intervals)
☒	☐	For null hypothesis testing, the test statistic (e.g. *F*, *t*, *r*) with confidence intervals, effect sizes, degrees of freedom and *P* value noted<br>*Give P values as exact values whenever suitable.*
☒	☐	For Bayesian analysis, information on the choice of priors and Markov chain Monte Carlo settings
☒	☐	For hierarchical and complex designs, identification of the appropriate level for tests and full reporting of outcomes
☒	☐	Estimates of effect sizes (e.g. Cohen's *d*, Pearson's *r*), indicating how they were calculated

Our web collection on statistics for biologists contains articles on many of the points above.

Software and code

Policy information about availability of computer code

Data collection	CFX Maestro Software (BioRad), ZEN Microscopy software
Data analysis	Precision Melt Analysis™ (BioRad), EthoVision XT 12 (Noldus), R v4.1.2, GraphPad Prism v8, ImageJ v2.3.0

For manuscripts utilizing custom algorithms or software that are central to the research but not yet described in published literature, software must be made available to editors and reviewers. We strongly encourage code deposition in a community repository (e.g. GitHub). See the Nature Portfolio guidelines for submitting code & software for further information.

Data

Policy information about availability of data

All manuscripts must include a data availability statement. This statement should provide the following information, where applicable:

- Accession codes, unique identifiers, or web links for publicly available datasets
- A description of any restrictions on data availability
- For clinical datasets or third party data, please ensure that the statement adheres to our policy

This study includes no data deposited in external repositories. No unbiased larger data sets were generated in this study. All data are presented in the results or as supplemental data. All unique zebrafish lines generated in the study are available with a completed materials transfer agreement. Any additional information required to reanalyze the data reported in this paper is available upon request.

Research involving human participants, their data, or biological material

Policy information about studies with human participants or human data. See also policy information about sex, gender (identity/presentation), and sexual orientation and race, ethnicity and racism.

Reporting on sex and gender	N/A
Reporting on race, ethnicity, or other socially relevant groupings	N/A
Population characteristics	N/A
Recruitment	N/A
Ethics oversight	N/A

Note that full information on the approval of the study protocol must also be provided in the manuscript.

Field-specific reporting

Please select the one below that is the best fit for your research. If you are not sure, read the appropriate sections before making your selection.

☒ Life sciences ☐ Behavioural & social sciences ☐ Ecological, evolutionary & environmental sciences

For a reference copy of the document with all sections, see nature.com/documents/nr-reporting-summary-flat.pdf

Life sciences study design

All studies must disclose on these points even when the disclosure is negative.

Sample size	The number of animals used in procedures was estimated based on reports in the current literature and consistent with the established standards.
Data exclusions	Outliers were excluded using a Grubbs' test ($p < 0.05$).
Replication	All experiments were performed at least twice and consistent results were observed between replicates.
Randomization	For experiments, zebrafish larvae grown in Petri dishes (with ~50 larvae per dish) were randomly selected.
Blinding	Experiments were not blinded, but the data analysis was performed using predefined criteria.

Reporting for specific materials, systems and methods

We require information from authors about some types of materials, experimental systems and methods used in many studies. Here, indicate whether each material, system or method listed is relevant to your study. If you are not sure if a list item applies to your research, read the appropriate section before selecting a response.

Materials & experimental systems

n/a	Involved in the study	
☐	☒	Antibodies
☒	☐	Eukaryotic cell lines
☒	☐	Palaeontology and archaeology
☐	☒	Animals and other organisms
☒	☐	Clinical data
☒	☐	Dual use research of concern
☒	☐	Plants

Methods

n/a	Involved in the study	
☒	☐	ChIP-seq
☒	☐	Flow cytometry
☒	☐	MRI-based neuroimaging

Antibodies

Antibodies used	Alkaline phosphatase-conjugated anti-DIG antibody (1:5000, Roche, Cat#11093274910)
Validation	It is commonly used in zebrafish for in situ hybridization in peer-reviewed literature (e.g Tessadori et al., eLife 2021).

Animals and other research organisms

Policy information about studies involving animals; ARRIVE guidelines recommended for reporting animal research, and Sex and Gender in Research

Laboratory animals	Zebrafish (Danio rerio) of mixed sexes, larvae under 5 days post-fertilization (dpf) or 8-14 month-old adult fish. mcu-/- and Tg(HuC:CEPIA2mt) lines were obtained from Prof. Jacek Kuźnicki laboratory. tmbim5-/- and slc8b1-/- lines were generated in-house as described in details in the manuscript.
Wild animals	The study did not involve wild animals.
Reporting on sex	The sex of the fish was not considered in the study, except for the analysis of the size and weight of adult fish, which only included male fish.
Field-collected samples	The study did not involve samples collected from field.
Ethics oversight	All experimental procedures were approved by the Local Ethical Committee for Experiments on Animals in Warsaw (permission no. WAW2/050/2022) and were performed in accordance with European and Polish regulations on animal welfare.

Note that full information on the approval of the study protocol must also be provided in the manuscript.

Plants

Seed stocks	N/A
Novel plant genotypes	N/A
Authentication	N/A
